# An insight into the commercial piglet’s microbial gut colonization: from birth towards weaning

**DOI:** 10.1186/s42523-022-00221-9

**Published:** 2022-12-26

**Authors:** Mireia Saladrigas-García, Mario Durán, Matilde D’Angelo, Jaume Coma, José Francisco Pérez, Susana María Martín-Orúe

**Affiliations:** 1 Servicio de Nutrición Y Bienestar Animal. Departamento de Ciencia Animal Y de los Alimentos, Universitat Autònoma de Barcelona, 08193 Bellaterra, Barcelona, Spain; 2 Grupo Vall Companys, 25191 Lleida, Spain

**Keywords:** Pig, Suckling piglet, Weaning, Microbiota, Colonization, Lactation, Gut health, Commercial farm

## Abstract

**Background:**

The establishment of the gut microbiota can be influenced by several perinatal factors, including, most importantly, the maternal microbiota. Moreover, early-life environmental variation affects gut microbial colonization and the intestinal health of offspring throughout life. The present study aimed to explore the development of piglet gut microbiota from birth to weaning in the commercial practice and also to assess how different farm environments could condition this process. Although it is possible to find in the literature other studies with similar objectives this work probably represents one of the few studies that make a systematic evaluation of such differential factors under a real scenario. To achieve this objective, we performed two trials. In a first Trial, we selected 2 farms in which we performed an intensive sampling (5 samples /animal) to characterize the gut colonization pattern during the first days of life and to identify the time window with the greatest impact. Both farms differed in their health status and the use of antimicrobials in the piglets. In a second Trial, we selected 4 additional farms with variable rearing conditions and a distinctive use of antimicrobials in the sows with a simplified sampling pattern (2 samples/animal). Faecal samples were obtained with swabs and DNA was extracted by using the PSP® Spin Stool DNA Kit and sequencing of the 16S rRNA gene (V3-V4 region) performed by Illumina MiSeq Platform.

**Results:**

The present study contributes to a better understanding of microbiome development during the transition from birth to weaning in commercial conditions. Alpha diversity was strongly affected by age, with an increased richness of species through time. Beta diversity decreased after weaning, suggesting a convergent evolvement among individuals. We pinpointed the early intestinal colonizers belonging to *Bacteroides, Escherichia-Shigella*, *Clostridium *sensu stricto* 1,* and *Fusobacterium* genera. During lactation(d7-d21 of life), the higher relative abundances of *Bacteroides* and *Lactobacillus* genera were correlated with a milk-oriented microbiome. As the piglets aged and after weaning (d36 of life), increasing abundances of genera such as *Prevotella*, *Butyricimonas*, *Christensenellaceae R-7 group*, *Dorea*, *Phascolarctobacterium*, *Rikenellaceae RC9 gut group*, *Subdoligranulum,* and *Ruminococcaceae UCG-002* were observed. These changes indicate the adaptation of the piglets to a cereal-based diet rich in oligosaccharides and starch. Our results also show that the farm can have a significant impact in such a process, evidencing the influence of different environments and rearing systems on the gut microbiota development of the young piglet. Differences between farms were more noticeable after weaning than during lactation with changes in alpha and beta biodiversity and specific taxa. The analysis of such differences suggests that piglets receiving intramuscular amoxicillin (days 2–5 of life) and being offered an acidifying rehydrating solution (Alpha farm in Trial 1) have a greater alpha diversity and more abundant *Lactobacillus* population. Moreover, the only farm that did not offer any rehydrating solution (Foxtrot farm in Trial 2) showed a lower alpha diversity (day 2 of life) and increased abundance of *Enterobacteriaceae* (both at 2 and 21 days). The use of in-feed antibiotics in the sows was also associated with structural changes in the piglets’ gut ecosystem although without changes in richness or diversity. Significant shifts could be registered in different microbial groups, particularly lower abundances of *Fusobacterium* in those piglets from medicated sows.

**Conclusions:**

In conclusion, during the first weeks of life, the pig microbiota showed a relevant succession of microbial groups towards a more homogeneous and stable ecosystem better adapted to the solid dry feed. In this relevant early-age process, the rearing conditions, the farm environment, and particularly the antimicrobial use in piglets and mothers determine changes that could have a relevant impact on gut microbiota maturation. More research is needed to elucidate the relative impact of these farm-induced early life-long changes in the growing pig.

**Supplementary Information:**

The online version contains supplementary material available at 10.1186/s42523-022-00221-9.

## Background

The process of microbial colonization of the gut after birth plays an important role in the development of the neonatal immune system of mammals with implications during their whole life [1]. Immediately after birth, environmental and maternal bacteria, including colonization via the vagina, nipple surface, and milk, quickly colonize the offspring gut and establish the initial microbiota of the piglet [2–4]. The intestinal microbiota protects against colonization by pathogens through bacterial competition and interaction [5]. The disruption of the healthy microbial community during the neonatal period may lead to the overgrowth of indigenous pathobionts and induction of pro-inflammatory status [6, 7]. It has been shown that stress, diet, management practices, and antimicrobial compounds during the early-life period may induce a long-lasting impact on the establishment of gut microbiota, disease susceptibility, and growth performances of offspring pigs [7–14]. This is especially relevant in swine production in which differences in husbandry and farm environment along the first days of life, could have a long-lasting impact on animal health and productive outcome. For instance, some researchers have reported how different exposure to stress or the use of antibiotics can determine changes in the gut microbial colonization of piglets 8 days after birth with implications in the immune development [11]. Evidence has also been published defining differences in the faecal microbiota of piglets as early as 7 days of life, determining their susceptibility to suffering post-weaning diarrhoea four weeks later [15], emphasizing the potential of the early microbiota establishment on the development of the immune response. It is, therefore, crucial to accurately determine the early-life development of the gut microbiome to eventually unravel microbiome effects on host phenotype and its impact on animal response and performance.

In commercial pig husbandry, weaning is an abrupt event comprising a dietary shift from sow milk to solid-feed-based diets, which poses a challenge to piglets during early-life development. During the pre-weaning phase, microbiome composition is dominated by a milk-oriented microbiome composed of families like *Bacteroidaceae* and *Lactobacillaceae* [16, 17], which rapidly changes after weaning when a solid cereal-based diet is introduced. For instance, butyrate-producing genera such as *Prevotella*, having a very low abundance in suckling piglets, dramatically increase post-weaning due to the availability of complex oligosaccharides and polysaccharides in the feed [16, 18, 19]. The rapidly changing microbiome of the young piglets seems to increase in diversity and richness along with the suckling phase and gradually stabilizes postweaning [16, 17, 20–22]. However, despite the recent advances in the knowledge of the development of intestinal microbiota in the young pig, there is still a lack of knowledge regarding how this pattern can be influenced by the farms' management practices and the microbiological environment in which piglets are raised.

Considering the great importance of the early gut microbiota development for pig future health and productive life, the objective of this work was, therefore, to determine the succession of microbial colonization that occurs in those piglets reared in commercial conditions and to identify possible pattern changes associated to different management guidelines and particularly to the use of antimicrobials. For that purpose, we performed two trials. In Trial 1, we selected 2 farms differing in their health status and the use of antimicrobials in the piglets performing an intensive sampling (5 samples /animal). In Trial 2, we selected 4 additional farms with a distinctive use of in-feed antibiotics in the sows and a simplified sampling pattern (2 samples/animal).

## Methods

### Animal ethics and experimental design

The experimental work was approved by the Animal Ethics Committee at the Autonomous University of Barcelona.

A longitudinal analysis of the development of the faecal microbiota of piglets reared under commercial conditions was carried out. A total of 6 farms (S1 and S2) from the same vertical integrator were selected, two were included in a first Trial and four in a second Trial. Each trial was performed at a different time of the year. The first Trial included an intensive sampling during the piglets’ first month of life approximately (5 time -points for each animal on days 2, 7, 14, 21, and 36 of life), and the second Trial a simplified sampling pattern (2 time-points for each animal on days 2 and 21). Stool samples were collected from the piglets by rectal swab and stored at room temperature until analysed. Piglets were a commercial crossbreed Piétrain × (Landrace × Large White) coming from multiparous sows. General information about the six farms involved in the study can be found in Table 1.

**Table 1:** Additional general information about each one of the six farms involved in the study

Farm ID	Trial 1		Trial 2			
	Alpha	Bravo	Charlie	Delta	Echo	Foxtrot
Number of sows^1^	1330 (10)	5144 (10)	3240 (15)	1800 (15)	518 (15)	2790 (15)
Production schedule	Weekly	Weekly	Weekly	Weekly	4-week period	Weekly
Biosafety level	High	High	High	High	High	High
Closed-cycle farm	No	No	No	No	No	No
Farm involved in an AB reduction program	Yes	No	No	No	No	No
Sow vaccination program	PRRS, porcine parvovirus, swine erysipelas, Aujeszky, Actinobacillus pleuropneumonia (APP), colibacillosis and rotavirus in gilts	PRRS, porcine parvovirus, swine erysipelas, Aujeszky, APP (toxoid), Mycoplasma hyopneumoniae, swine influenza, colibacillosis + clostridiosis, rotavirus in gilts and swine dysentery autovaccine	PRRS, porcine parvovirus, swine erysipelas, Aujeszky and colibacillosis	PRRS, porcine parvovirus, swine erysipelas, Aujeszky and colibacillosis	PRRS, porcine parvovirus, swine erysipelas, Aujeszky, colibacillosis and rotavirus in gilts	PRRS, porcine parvovirus, swine erysipelas, Aujeszky, swine influenza, colibacillosis and porcine circovirus
Recurring sanitary problems	No	APP and B*. hyodysenteriae*	No	No	*B. hyodysenteriae*	*B. hyodysenteriae*
Piglet processing at birth	Tail docking, iron administration by injection, oral administration of coccidiostat and antibiotic treatment (injected)	Tail docking, iron administration by injection and oral administration of coccidiostat	Tail docking, iron administration by injection, oral administration of coccidiostat and antibiotic treatment (injected)	Tail docking, iron administration by injection, oral administration of coccidiostat and antibiotic treatment (injected)	Tail docking, iron administration by injection, oral administration of coccidiostat and antibiotic treatment (injected)	Tail docking, iron administration by injection, oral administration of coccidiostat and antibiotic treatment (injected)
Regular use of creep-feeding	Yes	Yes	Yes	Yes	Yes	Yes
Use of AB in creep-feeding (including ZnO)	No	No	No	No	No	No
Regular use of re-hydrating solution during lactation	Yes	Yes	Yes	Yes	Yes	No
Piglet age at weaning	21 days	21 days	28 days	21 days	21 days	21 days
Cross fostering and litter management at birth	Nursing mothers at 24 h. Minimal movements	Equalization of litters trying to minimize movements. Piglets are collected only during the 1st week	Minimal movements	Minimal movements	Minimal movements	Minimal movements
Medicated maternal feed during lactation	No, NMF diet	No, NMF diet^2^	No, NMF diet	No, NMF diet	Yes, ABF diet^3^	Yes, ABF diet^2^

^1^The numbers in parentheses indicate the number of sows selected for the study

^2^Bravo farm sows routinely received a tulathromycin injection on day 16 postpartum (2.5 mg tulathromycin/kg BW)

^3^ABF diet consisted of the same NMF diet supplemented with 600 ppm of lincomycin

The two farms included in the first Trial (Alpha and Bravo farms) were selected based on their different health status and use of antimicrobials. Whereas Alpha could be considered a high-standard farm with a low incidence of pathologies, Bravo frequently coursed episodes of pleuropneumonia (*Actinobacillus pleuropneumonia*, APP) and swine dysentery (*Brachyspira hyodysenteriae*). Alpha was involved in an antibiotic reduction program and piglets received an intramuscular dose of amoxicillin (15 mg amoxicillin/kg BW) between their second and fifth day of life; moreover, they were given an oral rehydrating and acidifying solution the first week (Hidracid®, MEVET S.A.U, Lleida, Spain) including dextrose, sodium chloride, glycerine, monobasic potassium phosphate, mono, di and triglycerides of medium-chain fatty acids (C4, C8, and C10), potassium chloride and organic acids (formic acid, sodium formate, propionic acid, sodium citrate). In Bravo, piglets did not receive any antibiotic treatment, although the mothers received a tulathromycin injection on day 16 post-partum (2.5 mg tulathromycin/kg BW). The piglets were also given an oral rehydrating solution the 1st week of life (Hidravall®, MEVET S.A.U, Lleida, Spain) including dextrose, sodium chloride, monopotassium phosphate, and potassium chloride. In both farms, piglets were offered creep feed without zinc oxide. Mothers received a lactating non-medicated feed (NMF). After weaning the piglets were mixed according to the usual management procedures in each farm. The piglets were fed a feed without ZnO in all cases. In each farm, 10 litters were randomly selected, and one average piglet per litter was sampled. The same piglet was sampled on d2, d7, d14, and d21 of lactation and d14 after weaning (d36 of life), obtaining a total of 100 samples for Trial 1. However, in Alpha farm, the faecal sampling after weaning had to be anticipated due to an imminent antibiotic treatment of the piglets (d7 after weaning and d29 of life) because of a diarrhoea outbreak.

For the second Trial of the study, four farms were selected (Charlie, Delta, Echo and Foxtrot) trying also to analyse the possible impact of the use of antibiotics in the mothers’ diet. In two of them (Charlie and Delta) sows were fed NMF and in the other two (Echo and Foxtrot) sows received medicated feed with lincomycin (600 ppm) (ABF). Piglets from all farms were offered creep feed without ZnO and piglets from Charlie, Delta and Echo farms a rehydrating oral solution during the first week of life (Hidravall®, MEVET S.A.U, Lleida, Spain). In each farm 15 litters were randomly selected, sampling one piglet per litter. The same piglet was sampled on d2 and d21. Due to some casualties on d21 (by non-infections causes such as crushing, low viability or starvation) ultimately 102 paired samples (d2 + d21 from the same animal) were analysed. Information regarding the composition of lactation diets (NMF and ABF) is provided in Additional file 1: Table S1.

### Faecal DNA extraction and 16S rRNA sequencing

Stool samples were taken with the Stool Collection Tubes with DNA Stabilizer (STRATEC Molecular GmbH, Berlin, Germany). DNA was extracted from 1.4 mL of each stool-preserved sample using the PSP® Spin Stool DNA Kit (STRATEC Molecular GmbH, Berlin, Germany) according to the manufacturer's instructions following the optimization steps for bacterial DNA enrichment. DNA concentration and purity were verified with the NanoDrop ND-1000 spectrophotometer (NanoDrop Technologies, Wilmington, DE, USA.). For high-throughput sequencing of the faecal microbiota, the MiSeq® Reagent Kit V2 (500 cycles) (Illumina, San Diego, CA, USA) was used and the V3-V4 region of 16S rRNA was targeted. All subsequent steps were performed on the MiSeq Illumina instrument.

### Bioinformatics and statistical analysis

Raw sequencing reads (Fastq files) were independently processed, aligned and categorized using Divisive Amplicon Denoising Algorithm 2 (DADA2) [23], which was run as an R script (in R v.4.0.2) using its R package (dada2 v.1.16.0). Sequence reads were filtered using the recommended DADA2 parameters (that is, an expected error threshold of 2) and chimaeras were removed (“removeBimeraDenovo” command). Afterwards, sequences were processed into Amplicon Sequences Variants (ASV) at 99% of identity. ASV were classified to the lowest possible taxonomic level based using the SILVA reference database (v138) provided by the SILVA web service [24]. The diversity patterns within the ASV table were analysed using a custom bioinformatics pipeline implemented in R 4.0.2 (http://www.r-project.org). Support for DADA2 in R was achieved through the *phyloseq* package (v.1.32.0; available at https://joey711.github.io/phyloseq/) [25]. The alpha diversity metrics were calculated using the *phyloseq* “estimate_richness” function from the rarefied ASV tables and using the *microbiome* package (v.1.10.0) [26]. The observed species, the Chao1 index, the Simpson and inverse Simpson metrics and the Shannon diversity measures were estimated. For beta diversity, measurements were calculated using the Whittaker index [27] and the betadisper () function of the *vegan* package (v.2.5.6) [28] using relative abundances. To compare any differential effects, an ANOVA analysis was performed for alpha richness and diversity. The Non-metric multidimensional scaling (NMDS), analysis of similarities (ANOSIM), permutational analysis of variance (PERMANOVA) and the method of grouping of unweighted pairs with hierarchical grouping of arithmetic mean (UPGMA), all of them based on the distance of Bray–Curtis, were carried out to test the significance of differences in overall microbial composition. The normalization of the raw counts was performed using cumulative sum scaling (CSS) [29] and the differential abundance analysis was performed following the *metagenomeSeq* package (v.1.30.0) [30]. The taxa were aggregated at the phylum, family and genus levels and expressed as compositional data. Environmental (farm or administration of antibiotics to the mother (Trial 2)) and host-covariates (age) were all considered factors that might modulate the diversity, structure and profile of the microbial communities. All *p*-values from multiple testing were corrected with a false discovery rate (FDR) according to the procedure by Benjamini–Hochberg [31]. Differences were expressed as significant if adjusted p ≤ 0.05 or tendencies if 0.05 < *p* < 0.1. Results are presented as the mean ± the standard error (SE).

## Results

### Trial 1: intensive sampling in two farms with different health statuses

#### Changes in microbiota structure and biodiversity

An average of 55,190 ± 6,265 amplicon sequence variants (ASV) were obtained per sample, for a total of 100 samples of faecal content, with an increase in species richness as the piglets aged (*p* = 0.002). Higher richness values and reads were also detected in piglets from Bravo compared to Alpha farm (*p* < 0.001 and *p* = 0.007 for species richness and reads, respectively).

**Fig. 1:**
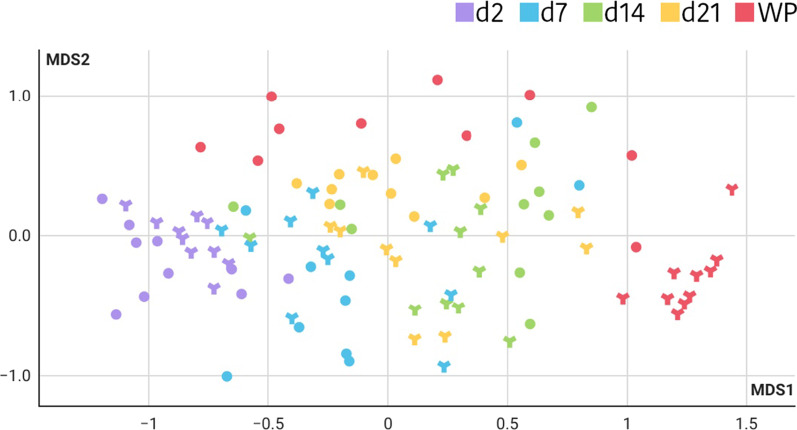
NMDS of the relative abundances of ASV in Trial 1. Different colours have been used to highlight each sampling day. The farm of origin is indicated with a different shape, round for the Alpha farm and with a 3-pointed star for the Bravo farm. A significant impact shaping the gut communities is produced by age and environmental factors (*p* < 0.001) with five clusters that match with piglet age. A greater richness is observed in Bravo piglets when compared to Alpha’s (*p* < 0.001), and with the increasing age of piglets (*p* < 0.001), suggesting that as the piglets grow and the gut community becomes more diverse, the divergence among animals increases. The significant effect of the farm is observed more clearly when performing the analysis by sampling day (Additional file 1. Figure S1)

Regarding changes in the ecosystem structure with age and environmental factors, PERMANOVA multivariate analysis showed that both, farm and animal age, showed significant effects in shaping the gut communities (*p* < 0.001). The non-metric multidimensional scaling (NMDS) based on the Bray–Curtis distance, showed five clusters that matched with piglet age (Fig. 1). The diversity indices also revealed important differences related to farms and ages. Following the richness results, greater alpha diversity was observed in Bravo farm and with the increasing age of piglets (*p* < 0.001), as presented in Additional file 1: Table S2. In addition, measured by the ANOSIM Bray–Curtis dissimilarities matrix, beta diversity also showed increased values with age (*p* = 0.001) suggesting that as the piglets grow and the gut community becomes more diverse, the divergence among animals increases.

To better analyse the impact of the farm, a complementary analysis was performed by sampling day. PERMANOVA within each sampled age (Additional file 1. Figure S1) showed a clear effect of the farm on community composition on all sampling days (*p* < 0.01). Along lactation differences between farms in alpha diversity (Additional file 1. Table S3) were significant at days 7 and 14 of life, showing Bravo farm higher values (*p* < 0.05 for all indices). However, no differences were found on day 21, probably due to the establishment of a more mature microbiota. Finally, after weaning a large impact of the farm of origin was observed (PERMANOVA, *p* < 0.001), in part due to the advanced sampling in the Alpha farm (7 days earlier than in the Bravo farm). Differences in the alpha and beta diversities between farms were also seen after weaning (*p* < 0.001).

#### Changes in taxonomic groups

**Fig. 2:**
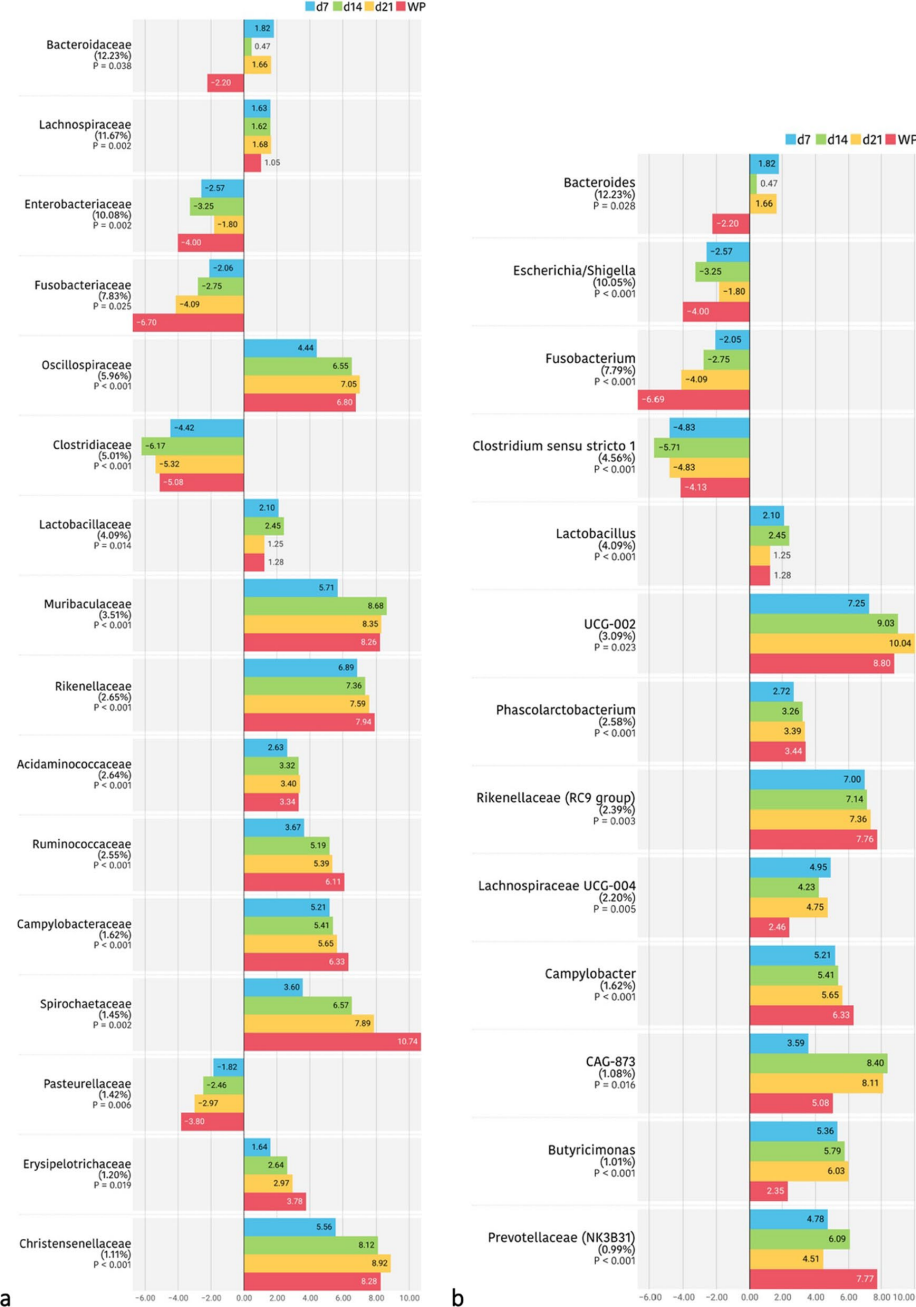
Differentially abundant taxa from faecal content (ln change and *p*-value < 0.05) among repeated samplings at family (**a**) and genus (**b**) level. Only predominant (> 1%) significant families and genera are presented; positive values and negative values indicate greater and lower abundance, respectively, in repeated samplings (d7, d14, d21 and weaned piglet) compared to new-born piglets (d2); taxa are sorted according to the general mean of relative abundance (the average of the entire trial, indicated between brackets in %) and in decreasing order. Figure created with the online open-source tool Datawrapper (http://datawrapper.de)

The temporal progression in the relative abundances of the main families is represented in Table 2 and Fig. 2. In the sampling closest to birth (day 2 of life), the microbiota of the piglets was composed of bacteria belonging to the phyla *Firmicutes* (36.98%), *Proteobacteria* (29.68%), *Fusobacteria* (18.34%) and *Bacteroidetes* (14.30%). Within the *Proteobacteria*, the main families observed were *Enterobacteriaceae* (24.62%), of which 10.30% were identified as *Escherichia/Shigella*, and *Pasteurellaceae* (4.50%). The phylum *Bacteroidetes* was represented by its main genus *Bacteroides* (11.89%), while the phylum *Firmicutes* was represented by a greater variety of families and genera, such as *Clostridiaceae* (18.56%), represented mainly by *Clostridium *sensu stricto* 1* (4.05%), *Lachnospiraceae* (7.87%), *Streptococcaceae* (3.41%) and *Lactobacillaceae* (1.97%), with a great relative abundance of its genus *Lactobacillus* (4.21%). Finally, the high presence of bacteria from the *Fusobacteriaceae* family (18.31%) and its genus *Fusobacterium* (10.04%) was found very characteristic of young piglets (d2 and d7).

**Table 2:**
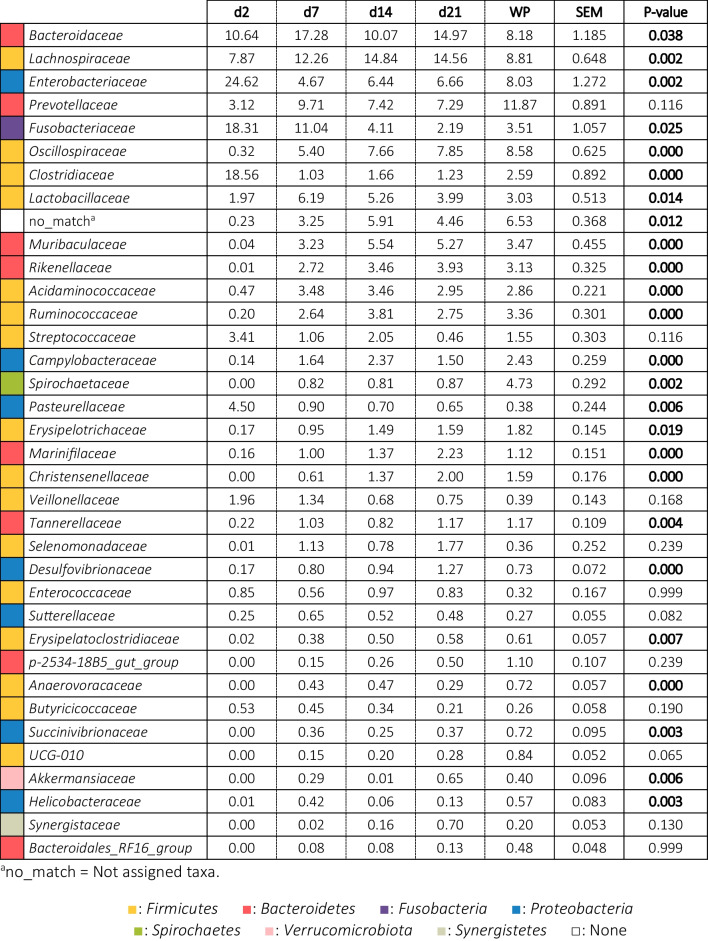
Relative abundances (%) of the families present in the faecal microbiota of the piglets in a percentage higher than 0.5%

Results came from 20 piglets sampled from two different farms during the first days of life. Faecal samples were collected on days 2, 7, 14 and 21 of lactation and 14 days after weaning (36 days of life). Results are presented in decreasing order of abundance concerning the general average. The colours represent the phyla to which the families belong. Significant changes are highlighted in bold.

At one week of life (d7), a large decrease in the relative abundance of the phylum *Proteobacteria* (6.89%) was observed, mainly due to the large decrease also observed in the *Enterobacteriaceae* family (4.67%), despite the fact that the relative abundance of *Escherichia/Shigella* genus level remained high (12.38%). There was a significant increase in the relative abundance of *Bacteroidetes* (35.64%), due to the increase in abundance of genera such as *Prevotella* (6.34%), *Rikenellaceae RC9 gut group* (1.94%) and *CAG-873* (1.82%). *Firmicutes* remained the predominant phylum (41.25%), although important changes were observed in its distribution, with increases in families such as *Lachnospiraceae* (12.26%), *Lactobacillaceae* (6.19%), *Oscillospiraceae* (5.40%) and *Acidaminococcaceae* (3.48%) and a large decrease in the *Clostridiaceae* family (1.03%), despite the fact that the relative abundance of the genus *Clostridium *sensu stricto* 1* remained high (5.21%). *Fusobacteria* decreased slightly (11.95%), although at genus level *Fusobacterium* continued to predominate (10.00%).

On day 14 and day 21, the microbial composition showed a more similar outcome to each other. *Firmicutes* and *Bacteroidetes* established themselves as the predominant phyla. The abundance of *Fusobacteria* dropped to a great extent (4.11% and 2.19% at d14 and d21, respectively) and *Proteobacteria* stabilized with a relative abundance at the phylum level of 10%. At this stage, there were no major changes in the families or genera present in the microbiota. Moreover, a higher evenness was observed, as the relative abundances of taxonomic groups seemed to equal each other in abundance, with less prevalence of specific genera as in the first days of life (evenness: 0.88 ± 0.043; 0.91 ± 0.027; 0.92 ± 0.024; 0.92 ± 0.036 and 0.95 ± 0.013 for day 2, 7, 14, 21 and WP respectively). Thus, higher abundances of bacteria belonging to *Firmicutes* were observed, such as *Lachnospiraceae* (14.84% and 14.56% at d14 and d21, respectively), *Oscillospiraceae* (7.66% and 7.85%), *Ruminococcaceae* (3.81% and 2.75%), *Erysipelotrichaceae* (1.49) % and 1.59%) and *Christensenellaceae* (1.37% and 2.00%). A slight decrease was also observed in the relative abundance of *Lactobacillaceae* (5.26% and 3.99% at d14 and d21, respectively), and in the *Bacteroidetes* families, such as *Bacteroidaceae* (10.07% and 14.97% at d14 and d21, respectively) and *Prevotellaceae* (7.42% and 7.29% at d14 and d21, respectively).

Finally, after weaning, a greater variety of species and evenness among them was observed. Firmicutes (43.69%) and Bacteroidetes (31.27%) were kept as predominant phyla, followed by Proteobacteria (15.50%). The Spirochaetes phylum increased significantly in this period, with a relative percentage of 4.74%. Fusobacteria maintained levels similar to those observed before weaning (3.52%). At the family level, an increase was observed with respect to the previous sampling in *Prevotellaceae* (11.87%) and *Spirochaetaceae* (4.73%), and a decrease in *Lachnospiraceae* (8.81%). At the genus level, a decrease in *Escherichia/Shigella* was observed with respect to the lactating piglets (5.31%).

**Fig. 3:**
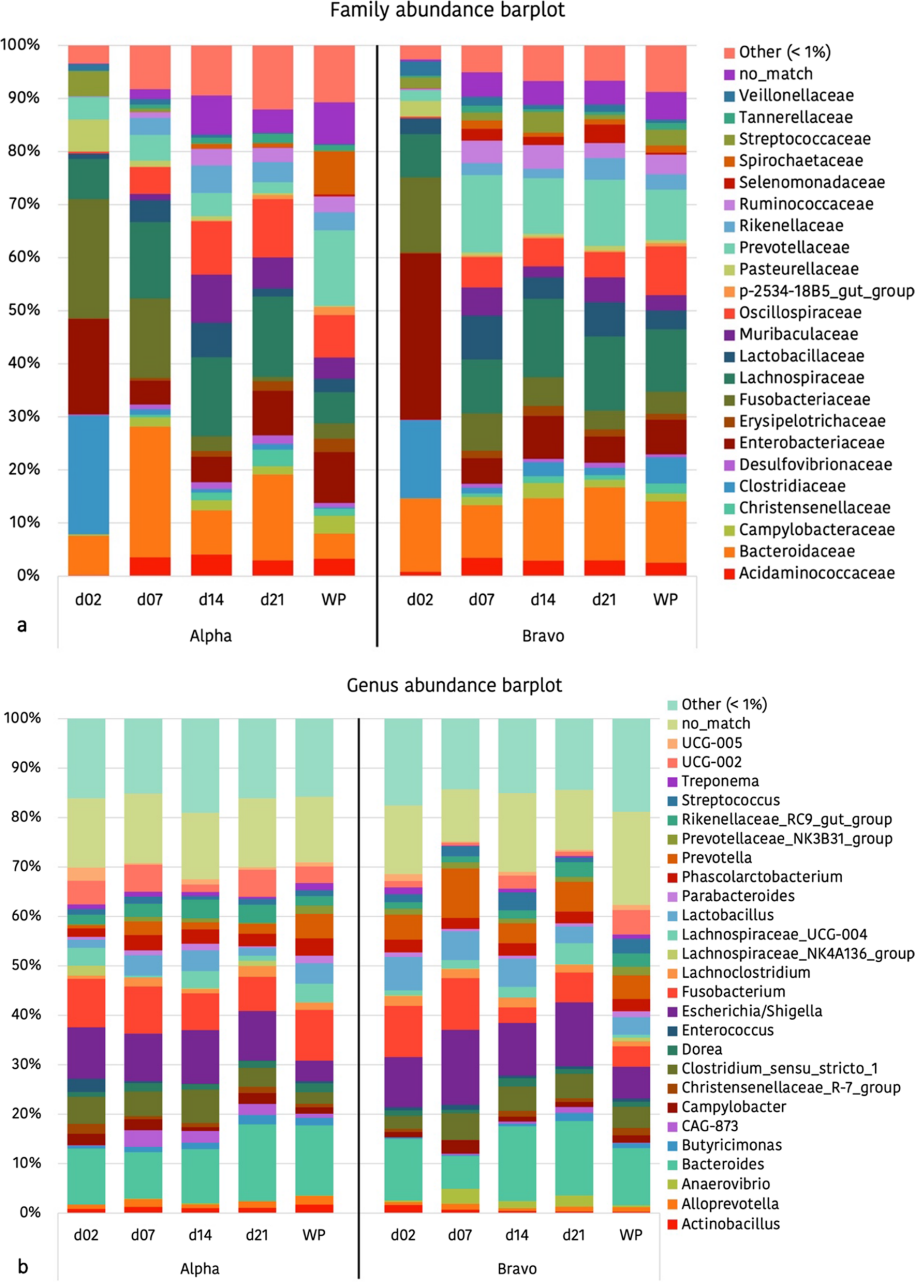
Bar diagram of the relative abundances (%) of the main (> 1%) families (**a**) and genera (**b**), organized by farm (Alpha or Bravo) and by age. WP = Weaned piglets

The effects of the farm of origin (Alpha or Bravo) in the relative abundances of main families and genera is represented in Fig. 3.

The taxonomic groups that showed significant differences between farms along the different sampling days can be found in Additional file 1: Figure S2 considering only the taxa detected in at least half of the samples. In general terms, it was observed that at day 2 of life and after weaning, the impact of the farm was greater than at days 7, 14 and 21 of life, since a higher number of differential taxa were detected. After weaning, differences among farms were manifested with significant changes in more than 19 genera. It is interesting to remark the lower numbers of *Enterobacteriaceae* and *Escherichia/Shigella* genera observed in Alpha farm on days 2 and 7 of life (2.9 and 3.4 negative ln change respectively) and the opposite pattern registered after weaning (3.2 positive ln change compared to Bravo farm).

More specifically and looking for differences between farms, we observed that at the phylum level and in 2-day-old piglets, *Proteobacteria* were increased in piglets from **Bravo** farm (*p* = 0.001). In parallel, the corresponding family and genus, *Enterobacteriaceae* and *Escherichia/Shigella*, respectively, were also enriched in **Bravo**-reared animals compared to the **Alpha**-reared animals (*p* < 0.001). Bacteroidetes was also found to be increased in piglets from the **Bravo** farm (*p* = 0.008), with the subsequent increase in its genus *Bacteroides* (*p* < 0.001). Some taxa belonging to the *Firmicutes* phylum were found enriched in piglets from the **Bravo** farm, such as the *Enterococcaceae* family (*p* < 0.001) and its genus *Enterococcus* (*p* < 0.001), and other genera such as *Lactobacillus* (*p* < 0.001), *Phascolarctobacterium* (*p* < 0.001) and *Clostridium *sensu stricto* 1* (*p* = 0.003). On the contrary, the *Actinobacillus* and *Dorea* genera obtained higher counts in the piglets from the **Alpha** farm (*p* = 0.002 and *p* = 0.016, respectively).

At one week of life (d7), the three predominant phyla: *Firmicutes*, *Proteobacteria* and *Bacteroidetes*, obtained higher counts in the piglets from the **Bravo** farm. This could be due to the higher counts observed in the *Enterobacteriaceae* family (*p* = 0.010) and its genus *Escherichia/Shigella* (*p* < 0.001); the family *Bacteroidaceae* (*p* = 0.043) and its genus *Bacteroides* (*p* < 0.001) and the various families belonging to *Firmicutes*: *Lachnospiraceae*, *Streptococcaceae* and *Ruminococcaceae* (*p* = 0.010, *p* = 0.016, and *p* = 0.030) and their genera *Streptococcus* (*p* < 0.001), *Lactobacillus* (*p* < 0.010), *Lachnoclostridium* (*p* = 0.015) and *Dorea* (*p* = 0.018), all of them, as stated before, with a higher presence in the piglets from the **Bravo** farm.

At two weeks of age (d14), the farm factor seems to have a lesser impact, with some differences between taxonomic groups, characterized mainly by a higher number in the piglets belonging to the **Bravo** farm of families belonging to the *Firmicutes* phylum, such as *Streptococcaceae* (*p* = 0.012) and *Lachnospiraceae* (*p* = 0.041) and the genera *Lachnoclostridium* (*p* = 0.005) and *Dorea* (*p* = 0.015). Similarly, higher counts of *Bacteroidetes* are observed in piglets reared on the **Bravo** farm, coinciding at the family level with *Prevotellaceae* (*p* = 0.019) and its genus *NK3B31 group* (*p* = 0.037).

At day 21 of life, just before weaning, a great significance is observed in the differential abundance of *Fusobacteria* (*p* = 0.016), with higher counts in the piglets reared on the **Bravo** farm either at the level of phylum, family (*p* = 0.019) or gender (*p* = 0.017). Likewise, lower *Lactobacillus* counts were detected in piglets reared on the **Alpha** farm (*p* = 0.046).

After weaning great shifts were observed. *Fusobacteriaceae* and *Enterobacteriaceae*, and their respective genera, *Fusobacterium* and *Escherichia/Shigella*, were significantly more enriched in piglets reared on the **Alpha** farm (*p* = 0.010 and *p* < 0.001 for *Fusoacterium* and *Escherichia/Shigella*, respectively). On the contrary, various families and genera belonging to the *Firmicutes* phylum were found enriched in the piglets reared on the **Bravo** farm. For instance, within the *Lachnospiraceae* family, there were higher counts of *Coprococcus* (*p* < 0.001), *Lachnospiraceae* UCG-004 (*p* = 0.004), *Lachnoclostridium* (*p* = 0.009) and *Dorea* (*p* = 0.028). Similarly, in piglets from the **Bravo** farm, higher counts of *Streptococcus* (*p* < 0.001), *Faecalibacterium* (*p* < 0.001), *Phascolarctobacterium* (*p* = 0.008), and *Lactobacillus* (*p* = 0.021), among others, were observed with respect to the piglets from the **Alpha** farm. Finally, the *Bacteroidetes* phylum was also found to be enriched in piglets from the **Bravo** farm, with higher counts of genera such as *Prevotella* (*p* < 0.001), *Prevotellaceae NK3B31 group* (*p* = 0.002), *Rikenellaceae RC9 gut group* (*p* = 0.002) and *Parabacteroides* (*p* = 0.036). Exceptionally, the *Bacteroides* genus was found to be more enriched in the piglets from the **Alpha** farm (*p* = 0.019).

### Trial 2: impact of the farm management practices on piglets’ microbiota

#### Changes in microbiota structure and biodiversity

An average of 74,429 ± 3,611 amplicon sequence variants (ASV) were obtained per sample for a total of 102 samples of faecal content. As found in the previous trial, a greater richness was observed in 21-day-old piglets when compared to 2-day-old piglets (*p* < 0.001). Among the different farms, no difference was detected in terms of richness, although a higher number of reads was observed in the Foxtrot farm (*p* = 0.057).

**Fig. 4:**
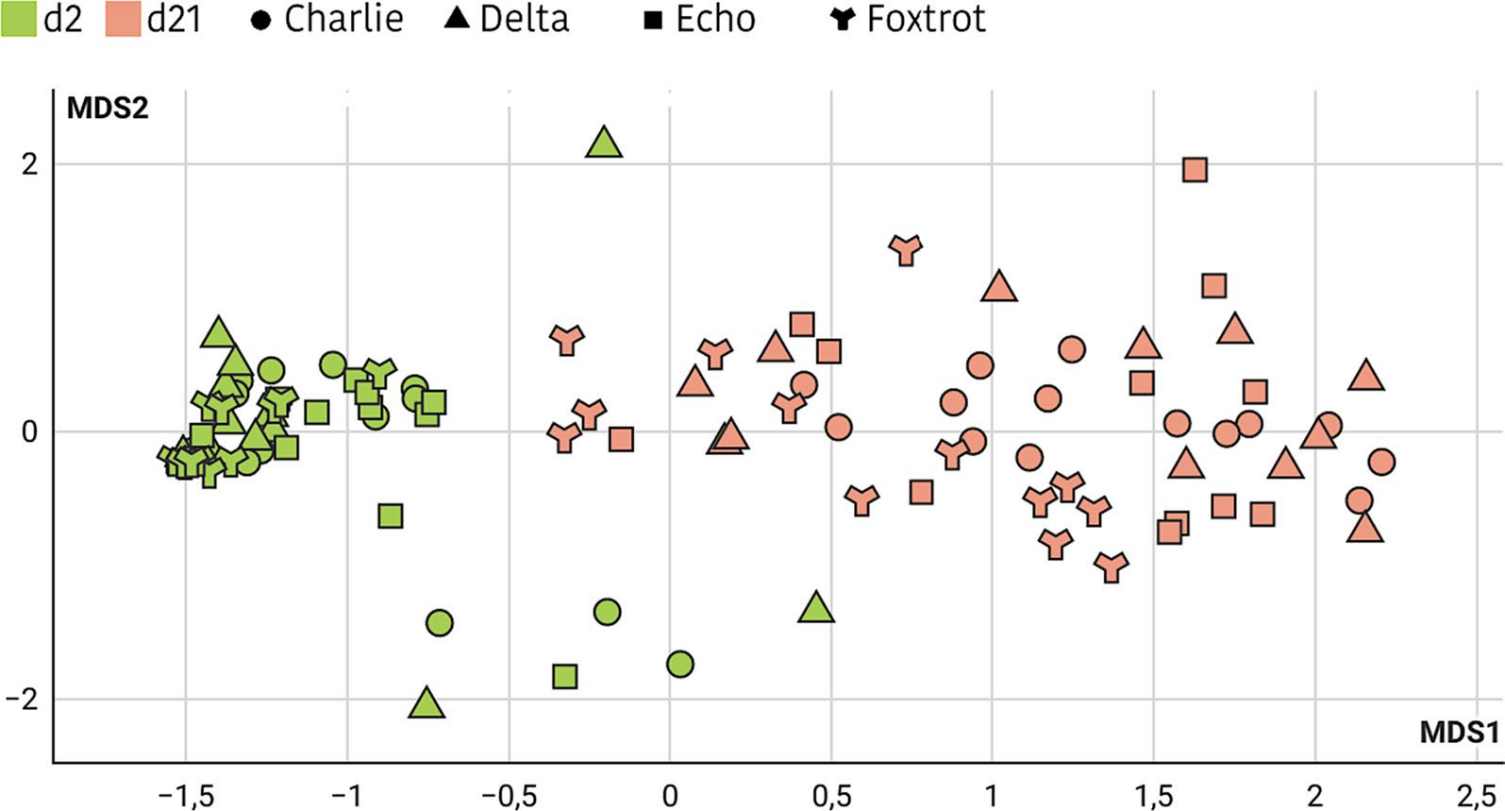
NMDS of the relative abundances of ASV in Trial 2. Different colours have been used to highlight each sampling day and different shapes to distinguish each farm. A significant impact shaping the gut communities is produced by the piglets’ age (*p* < 0.001) with two clear clusters between 2-day-old piglets and 21-day-old piglets. A greater richness was observed in 21-day-old piglets when compared to 2-day-old piglets (*p* < 0.001). Farm and medicated feed were not found to be significant by the NMDS analysis

Regarding changes in the microbial ecosystem with age, farm environmental factors and the use of medicated feed in the sows, PERMANOVA multivariate analysis showed that all factors considered (age, farm and medicated feed) had a significant impact on shaping the gut communities (*p* < 0.001 for age factor and *p* = 0.001 for farm and feed). However, the non-metric multidimensional scaling (NMDS) based on the Bray–Curtis distance, only found significant the effect of age (*p* < 0.001) with two clear clusters between 2-day-old piglets and 21-day-old piglets (Fig. 4).

**Fig. 5:**
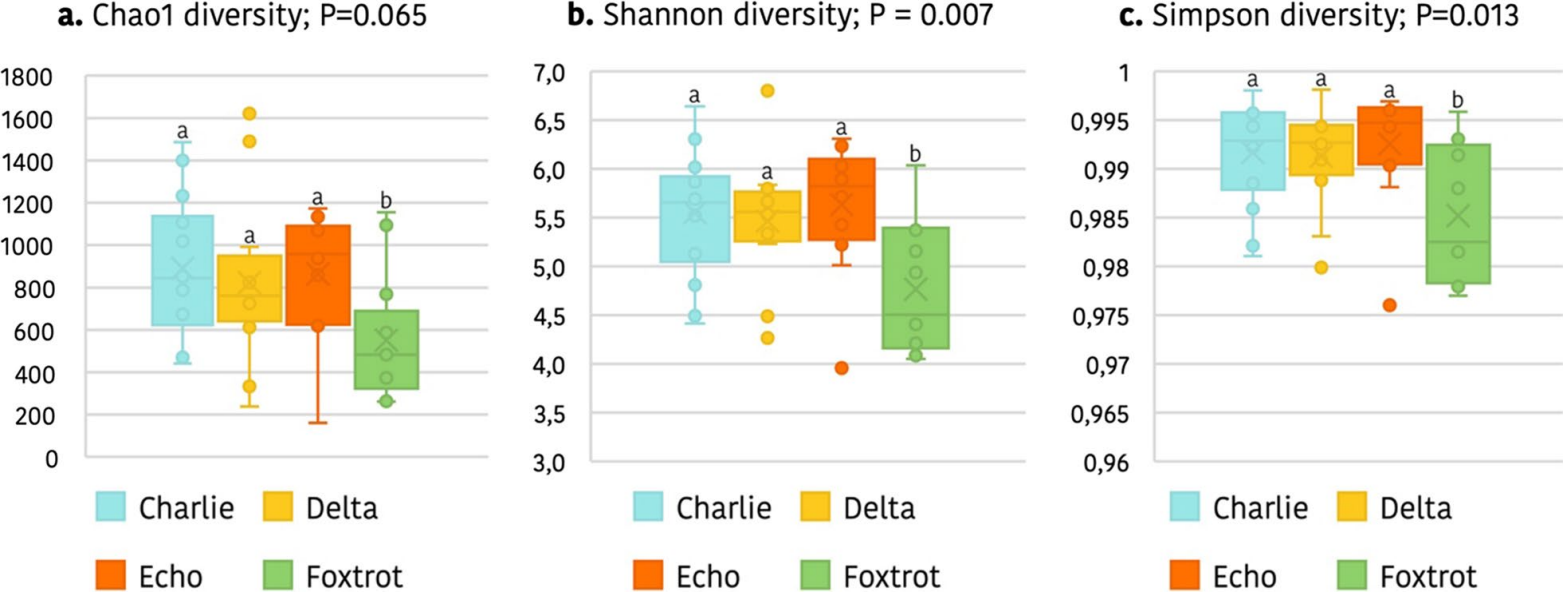
Box plot of the alpha diversity calculated using the Chao1 (**a**), Shannon (**b**) and Simpson indices (**c**) of the four farms included in Trial 2 in 2-day-old piglets. The Foxtrot farm showed lower alpha diversity values than the rest of the farms, suggesting a possible effect of maternal medicated feed

The diversity indices also revealed important differences among farms, use of medicated feed in sows, and piglet age. In accordance with the richness results, greater alpha diversity was observed in 21-day-old piglets compared to day 2, as presented in Additional file 1: Table S4. Moreover, Delta and Foxtrot farms were found to have significantly lower alpha-diversities for Observed species, Chao1 and Shannon indices than Charlie and Echo (*p* = 0.033, *p* = 0.034, and *p* = 0.027, respectively for farm effect) despite at day 2 of life decreases in alpha-diversity were only found in Foxtrot farm (Fig. 5). No significant impact of the use of medicated diet was found.

Regarding beta diversity calculated by means of the Whittaker index, no differences were found, neither between the sampling days, nor among farms or the use of medicated feed in sows. However, the multivariate analysis of ecological communities by the ANOSIM Bray–Curtis dissimilarities matrix, showed a significant effect of farm (*p* = 0.011 and *p* = 0.001, for d2 and d21, respectively) and of use of medicated feed in sows (*p* = 0.011 and *p* = 0.001, for d2 and d21, respectively). In 2-day-old piglets, a greater similarity was observed among the samples from the Foxtrot farm, while the rest of the farms presented similar results among them. However, on the 21st day of life, it was the Charlie farm that stood out for its greater similarity among samples. Regarding the use of medicated feed in mothers, in 2-day-old piglets, a greater similarity was observed among the microbiota of the piglets from medicated mothers (ABF), while on day 21 the piglets from the non-medicated groups (NMF) showed a greater similarity among each other.

#### Changes in taxonomic groups

**Fig. 6:**
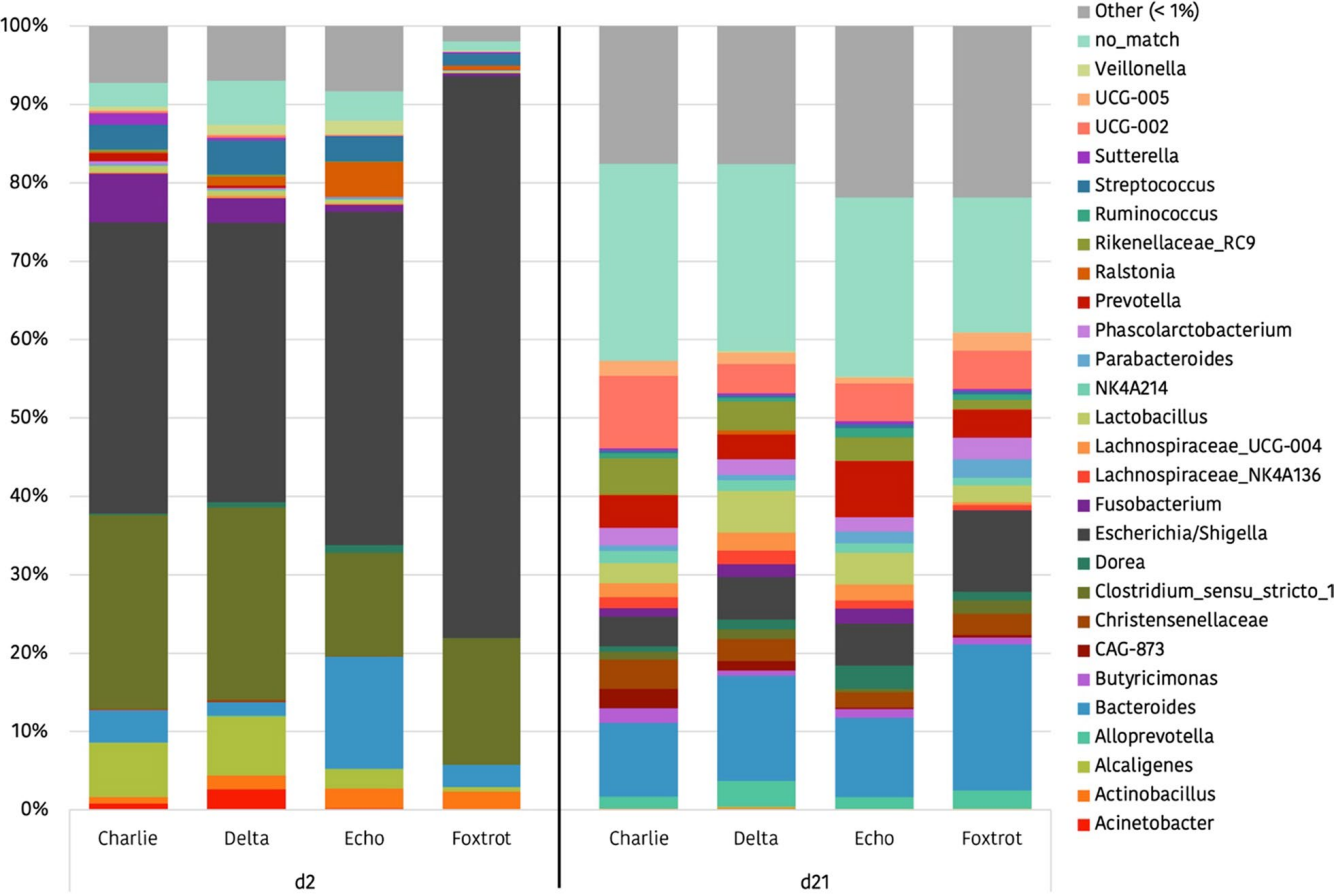
Bar diagram of the relative abundances (%) of the main genera (> 1%) in Trial 2, organized by farm (Charlie, Delta, Echo or Foxtrot) and by sampling day. In Echo and Foxtrot farms the sows received medicated feed

*Proteobacteria* and *Firmicutes* constituted the two predominant phyla in the faecal microbiota of 2-day-old piglets, contributing with a 58.0% and 30.6% of the relative abundance, respectively. *Bacteroidetes* (7.1%) and *Fusobacteria* (2.8%), with a representation greater than 1% in relative abundance, were also considered predominant phyla. At day 21 of life, a very different scenario was observed, with *Firmicutes* and *Bacteroidetes* being the two predominant phyla in the faecal microbiota of the piglet, contributing 49.19 and 35.73% of the relative abundance, respectively. *Proteobacteria* (9.73%) and *Fusobacteria* (1.14%), were also considered predominant phyla. Changes at genus level with the age of the pig were also remarkable and are shown in Fig. 6. From a total of 476 genera detected only 27 taxa represented a relative abundance greater than 1%.

**Fig. 7:**
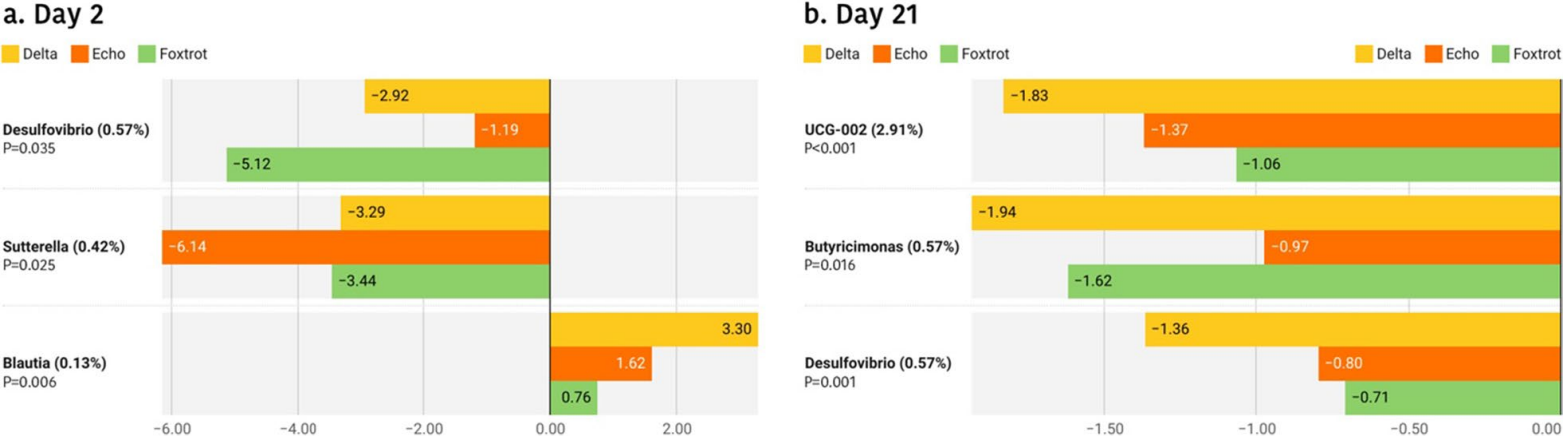
Differentially abundant taxa from faecal content (ln change and *p*-value < 0.05) among farms at genus level in 2-day-old (**a**) and 21-day-old (**b**) piglets. Only predominant (> 0.5%) significant genera are presented; positive values and negative values indicate greater and lower abundance, respectively, among farm environments (Delta, Echo and Foxtrot) compared to Charlie farm; taxa are sorted according to the general mean of relative abundance (the average of the entire trial, indicated between brackets in %) and in decreasing order. Figure created with the online open-source tool Datawrapper (http://datawrapper.de)

Regarding the possible effects of the farm on microbial taxonomy, Tables 3 and 4 show the relative abundances of the predominant families (> 0.5%), in the different farms, on days 2 and 21 respectively, and Fig. 7 shows differences in the genus level. Two days after birth, significantly higher levels for the *Bacteroidaceae* family were detected at the Echo farm. *Moraxellaceae* family was the lowest in the Foxtrot and the highest in the Delta farm. Trends were detected for other families, with remarkably higher levels (*p* = 0.059) of *Enterobacteriaceae* in the Foxtrot farm (Table 3). At the genus level, *Sutterella*, *Desulfovibrio* and *Blautia* also showed different abundance among farms (*p* = 0.025; *p* = 0.035 and *p* = 0.006 respectively) (Fig. 7). At day 21 of life, significantly lower levels of *Lachnospiraceae* and *Fusobacteriaceae* and higher levels of *Enterococcaceae* were observed in the Foxtrot farm (Table 4). At genus level *UCG-002*, *Desulfovibrio* and *Butyricimonas* also showed different abundance among farms (*p* < 0.001; *p* = 0.016 and *p* = 0.001, respectively) (Fig. 7).

**Table 3:**
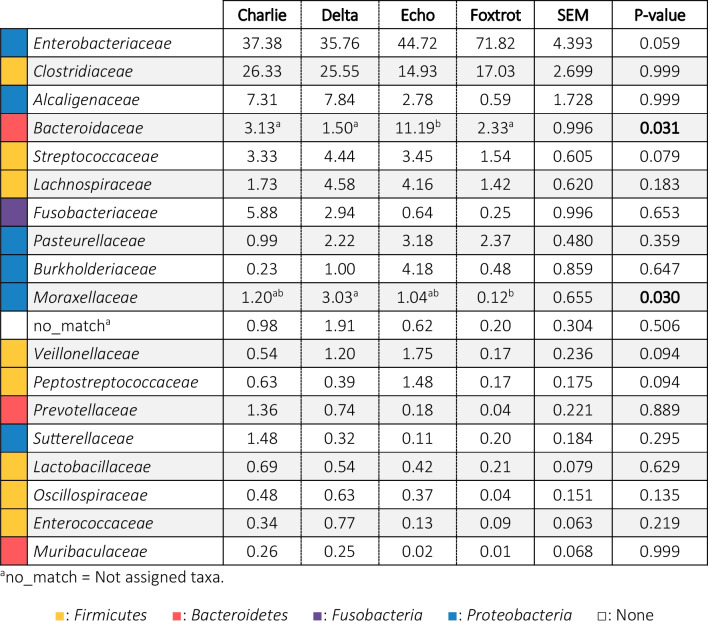
Relative abundances (%) of the families present in the faecal microbiota of 2-day-old piglets from Trial 2 in a percentage higher than 0.5%, classified by farm and in decreasing order of abundance in relation to the general average

The colours represent the phyla to which the families belong. In Echo and Foxtrot farms, sows received medicated feed; in Charlie, Delta and Echo farms piglets were offered a rehydrating solution during lactation. Significant changes are highlighted in bold.

**Table 4:**
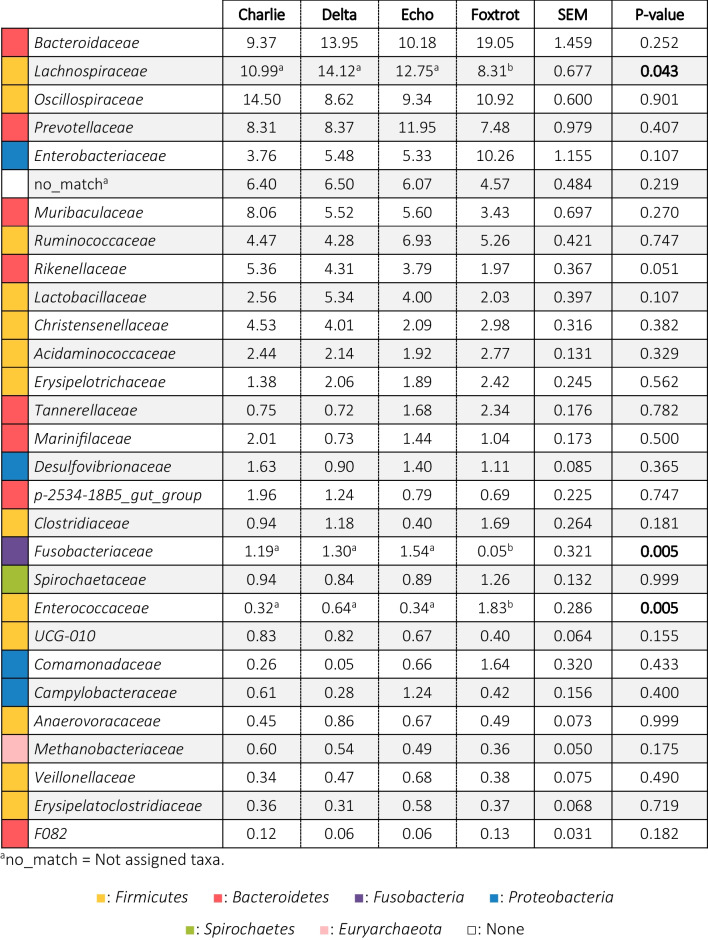
Relative abundances (%) of the families present in the faecal microbiota of 21-day-old piglets from Trial 2 in a percentage higher than 0.5%, classified by farm and in decreasing order of abundance in relation to the general average

The colours represent the phyla to which the families belong. In Echo and Foxtrot farms, sows received medicated feed; in Charlie, Delta and Echo farms piglets were offered a rehydrating solution during lactation. Significant changes are highlighted in bold.

**Fig. 8:**
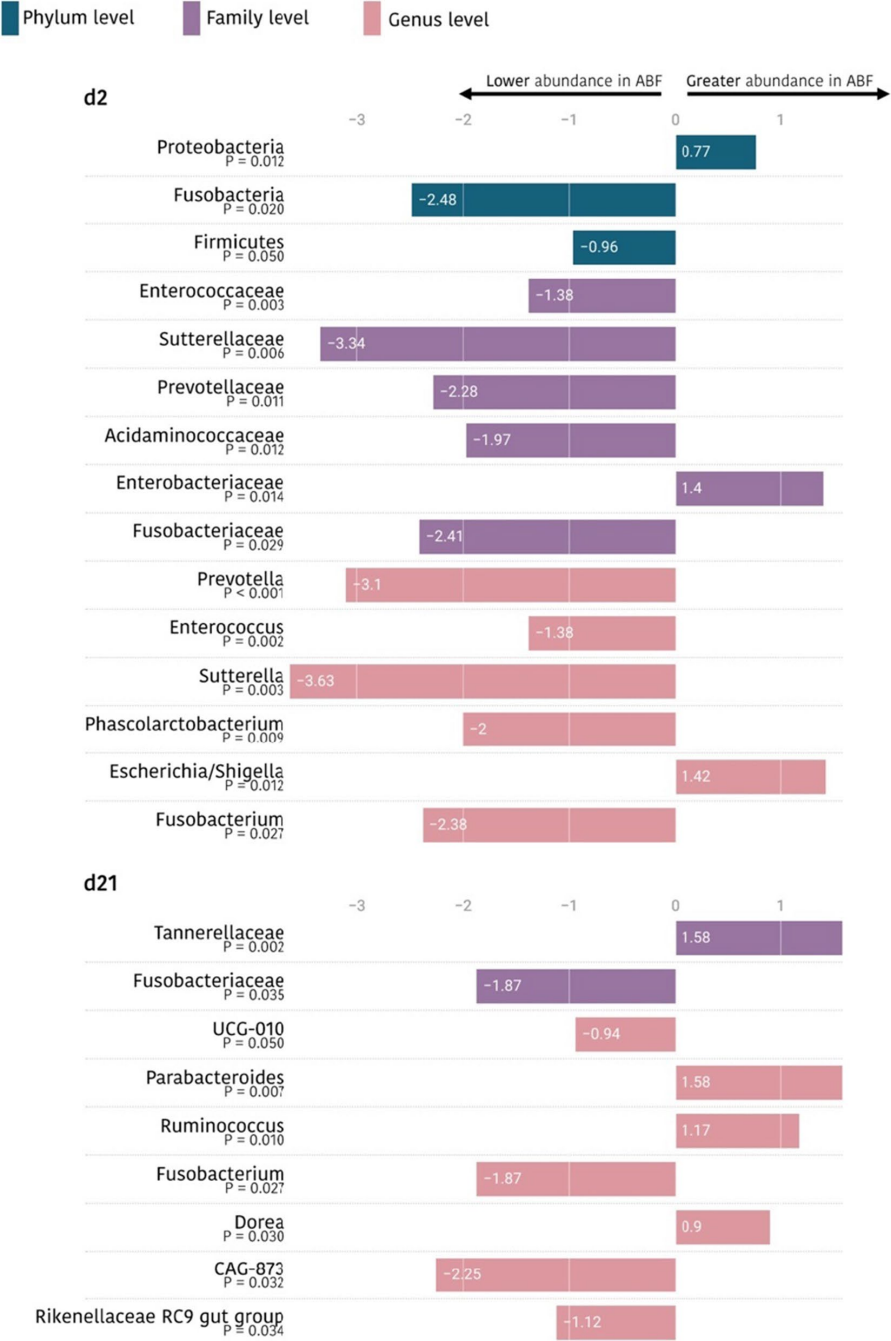
Ln changes in taxa promoted by sow feed (sows receiving (ABF) vs not (NMF) medicated feed; ln change and *p*-value < 0.05) at phylum, family and genus level in the microbiota of piglets. Positive values and negative values indicate higher and lower abundance, respectively, in piglets from ABF-fed mothers. Taxa are sorted by level of significance (from higher to lower). Only taxa with relative abundances higher than 0.5% are included in the figure. The presented differences are based only on taxa detected in at least half of the animals per sampling. Figure created with the online open-source tool Datawrapper (http://datawrapper.de)

Regarding the possible impact of the use of medicated feed in the microbiota of the progeny, Fig. 8 shows those taxonomic groups (families and genera) that were significantly modified at days 2 and 21 of life. At day 2 of life, piglets from dams fed non-medicated feed (NMF) showed statistically significant lower abundances of Firmicutes and Fusobacteria phyla explained by the lower detected levels of *Fusobacteriaceae*, *Prevotellaceae*, *Sutterellaceae*, *Enterococcaceae* and *Acidaminococcaceae* families. Regarding genera, *Fusobacterium*, *Prevotella*, *Phascolarctobacterium*, *Sutterella* and *Enterococcus* also showed significant lower abundances. Moreover, NMF farms were associated with higher counts of the *Enterobacteriaceae* family and the *Escherichia/Shigella* genera. On day 21 of life, changes were only observed in minor taxonomic groups (Fig. 8).

## Discussion

The main findings of this study include a detailed description of the gut microbial colonization pattern in the commercial piglet and a first analysis of the potential impact of the farm husbandry guides on such process. Most studies on pig microbiota have been conducted under standardized experimental conditions, and from this point of view, this is probably one of the few studies performed in real farms where the situation differs largely. In the following sections, we will discuss the possible definition of the gut microbial colonisation process in the piglet and those management or environmental variables capable to condition such pattern.

### The pattern of microbial colonization during the first weeks of life

After birth, the intestinal microbiome of the piglet rapidly undergoes a remarkable shift from the initial microbial groups which are present during the first days of life to the establishment of an adult-like microbial community, experiencing in between a period of changing microbial successions [12, 32, 33]. This initial pattern of microbial maturation is essential as the gut microbiota is fundamental for the adequate development and programming of the mucosal immune response [13]. Moreover, a “window of opportunity” has been described during this early-life development stage, where any disruption may have long-lasting impacts on health and welfare [34]. Therefore, understanding the dynamics of the young piglet gut microbiota during the first weeks of life is certainly concerning as its a major impact on the future health and growth performance of pigs.

Several authors have affirmed that age and weaning are the driving factors of microbiota development, pointing out specific changes in specific taxonomic groups at certain ages [16, 20–22, 32, 35, 36]. Initially, the gut colonization of newborn piglets is characterized by bacteria belonging to the *Clostridiaceace*, *Enterobacteriaceae*, *Fusobacteriaceae*, and *Bacteroidaceae* families, which are progressively replaced by carbohydrate fermenting bacteria, essentially the acetate, propionate, and butyrate-producing microorganisms. During the weaning transition, the microbial ecosystem evolves from a microbiome oriented to the degradation of milk carbohydrates, composed of families like *Bacteroidaceae* and *Lactobacillaceae*, towards a more intricate one.

Finally, microorganisms belonging to the *Lachnospiraceae* and *Ruminococcaceae* families, adapted to metabolize a wide range of complex oligosaccharides and polysaccharides while producing SCFA, have been reported to begin to emerge after weaning [17, 37].

For instance, the gradual increase observed in the microbial diversity of piglets as they aged, in the present study, is in accordance with several previous works [16, 17, 21, 22, 32, 36, 38–40]. A higher diversity in the gut microbiota has been related to a more mature gut ecosystem and is in agreement with the concept of functional redundancy, which supports that additional taxa add redundancy to specific functions, helping the ecosystem to preserve its resilience and stability after environmental stress [22, 41–43]. Diversity results are, however, contradictory with some other studies that have reported a decreased alpha diversity during the early period after weaning [37, 44, 45], with a later increase from weaning to adulthood. This discrepancy might be due to differences among studies in weaning age or the post-weaning sampling time-point but also to differences in management and feeding practices or in the microbiological environment of the farm. Regarding variability of the microbiota among individual piglets (interindividual Bray–Curtis distances), several authors have described that it tends to diminish as the piglets age [17, 22, 40, 46], suggesting that gut microbiota structure of piglets tends to converge among animals as they age to establish of a homogenous, rich and stable microbiota composition after weaning. However, results from the present work showed that this evolution is not always present, as in Trial 1 beta diversity was increased as the piglets grew. It is possible that under the complex and challenging environmental conditions the animals have to face in commercial practice, the evolution of their gut ecosystems will diverge more widely than when they are reared under highly standardized experimental conditions.

Firmicutes followed by Bacteroidetes were found to be the dominant phyla across all experimental samplings except for 2-day-old piglets. Although this is consistent with the majority of earlier studies [16, 19, 20, 22, 32, 36, 39], there are a few reports with Bacteroidetes or Proteobacteria [21, 47] as the pre-dominant phyla. However, the age of the piglet plays an important role in this result. In the present study, we observed that in 2-day-old piglets from Trial 1 Proteobacteria was found to be almost as abundant as Firmicutes and in Trial 2, Proteobacteria doubled Firmicutes in relative abundance. Therefore, the Proteobacteria phylum stands out in this study for its important weight at the time closest to birth. The decreasing abundance of Fusobacteria observed in Trial 1 during the first weeks of life has also been reported by several other authors [17, 21, 32, 38–40]. However, some studies have not reported the presence of this bacterial group at all [16, 35]. This is in consonance with the results observed in Trial 2, where much lower abundances than those previously reported in Trial 1 were detected. Such differences in taxonomic abundance could, to some extent, be due to variability factors such as the study design and conditions, pig genetics and environmental factors including the time of the year the sampling was performed, the sampling procedures or the analytical procedures. A trend towards higher abundance was observed for the phyla *Spirochaetes* as the piglets aged in both Trials, as observed by Pajarillo et al. [32]. At the family level, relative abundances of *Enterobacteriaceae*, *Clostridiaceae*, *Fusobacteriaceae*, *and Veillonellaceae* declined over time while those of *Prevotellaceae*, Ruminococcaceae, *Spirochaetaceae*, *Rikenellaceae*, *Erysipelotrichaceae* and *Succinovibrionaceae* increased. Similar results have also been reported in previous studies [20, 32, 33, 46]. However, contrary to what was reported by these authors, we did not observe a decrease in the *Bacteroidaceae* family in Trial 1 or an increase in the *Veillonellaceae* family. *Bacteroidaceae*, *Lactobacillaceae*, *Lachnospiraceae* and *Streptococcaceae* families showed oscillations in their relative abundances throughout the repeated samplings, generally increasing during lactation and slightly decreasing after weaning.

The present study pinpointed the early intestinal colonizers belonging to *Bacteroides, Escherichia-Shigella*, *Clostridium *sensu stricto* 1* and *Fusobacterium* genera. This is in accordance with Petri et al. [48], who reported the genera *Escherichia, Clostridium, Fusobacterium, Streptococcus*, and *Enterococcus* to be the earliest colonizers of the pig gut, between birth and 2 days. However, a high level of individuality has been reported to occur in 1- and 2-week-old piglets, indicating that there is considerable randomness to the process of acquiring microbes [8]. Although this gut community in very young piglets might be highly dynamic, the microbial community is known to stabilize by day 28. Decreases in the abundances of *Clostridium*, *Fusobacterium* and *Escherichia-Shigella* with the age of the piglets have also been observed by several other authors [16, 17, 19, 22, 32, 46]. During lactation, the genera *Bacteroides* and *Lactobacillus* acquire greater relative importance with respect to the rest of the genera and this fact has been correlated with a milk-oriented microbiome [16]. *Bacteroides* has been reported to use a wide range of both milk oligosaccharides and host-derived glycans [49] and *Lactobacillus* is a well-known lactate producer from lactose [50]. Moreover, whereas *Fusobacterium* has been positively correlated to intestinal diseases [51–53], *Lactobacillus* has been labelled as a major player in the establishment and maintenance of bacterial homeostasis after birth [2].

The second significant change in the microbial community of the piglet occurs in the period around weaning. In the present study, the increase in genera such as *Prevotella*, *Butyricimonas*, *Christensenellaceae R-7 group*, *Dorea*, *Phascolarctobacterium*, *Rikenellaceae RC9 gut group*, *Subdoligranulum* and *Ruminococcaceae UCG-002* stands out. This is largely in agreement with previous observations which exemplify *Prevotella* as a prominent actor in the typical post-weaning microbiota together with species belonging to *Roseburia, Faecalibacterium, Ruminococcus*, *Lachnospira*, *Dorea*, *Blautia, Subdoligranulum* [16, 17, 19–21, 32, 33, 35, 37, 40, 46, 54]. The drastic increase in the relative abundance of *Prevotella* is likely due to the established capacity of the members of this genus to metabolize plant-derived non-starch polysaccharides to short-chain fatty acids (SCFAs) [55, 56], which are prominent constituents of the cereal-based weaner diet. Therefore, the abrupt change to a solid cereal-based diet and the withdrawal of milk would explain the decrease of the previously mentioned genera and the increase of propionate- and butyrate-producing genera. Altogether, the higher abundance of propionate- and butyrate-producing genera in older piglets, adapted to digest resistant starches and dietary fibres, show the quick microbial transformation of the piglets’ gut microbiota to cope with diets rich in complex carbohydrates.

### The impact of farm management and environment on gut microbial colonization of piglets

In the present study, 6 commercial farms were included in order to determine the extent to which variations in the farm environment and rearing conditions influence the microbiota development after birth. Farm variability was relatively controlled since all the selected farms were indoors, close-cycle, geographically located in the same region of Spain, and vertically integrated using the same breed and feed formula. Moreover, farms included in Trial 1 or Trial 2 were sampled in the same temporal period. Differences among farms were mostly due to the metaphylactic use of antimicrobials (injected after birth to piglets and/or given to the sows as medicate feed) and rehydrating solutions during lactation. Farms were also selected based on differences in historical records of digestive (*B. hyodysenteriae*) and respiratory (*Actinobacillus pleuropneumonia*, APP) diseases (see Table 1 for additional information). After Trial 1, days 2 and 21 were chosen as the most relevant sampling days in Trial 2 to characterize the evolution pattern of the gut microbial ecosystem after birth and to assess possible factors influencing this process. Day 2 would reflect one of the moments of greater variability in the establishment process of the first colonizers and day 21 would give a relatively accurate picture of the first ecological equilibrium reached by the gut microbiota after birth.

As expected, significant differences among farms were found in the microbial ecosystem of piglets in both trials. In Trial 1, greater alpha diversity was found in the Bravo farm, whereas in Trial 2, lower alpha diversity was observed in the Foxtrot farm in 2-day-old piglets. This difference among farms was also demonstrated by the fact that several taxa were influenced by the rearing farm. Although the impact of the effect of the rearing farm on specific taxa has not been analysed in detail in the literature, changes in the microbiota of the piglets have been reported [46, 57]. Indeed, in addition to animal age and genetic background [58], the structure and activity of gut microbiota can differ among animals depending on various factors including dietary influence [16, 59, 60], sanitary status [61], antimicrobial use [14, 62–64] and husbandry practices [65, 66], among others. For instance, in Trial 1, piglets from Alpha farm received an intramuscular dose of amoxicillin (15 mg amoxicillin/kg BW) between their second and fifth day of life, which could explain the lower alpha diversity found in Alpha piglets. Although most of the existing literature has focused on the study of oral administration of antibiotics, a recent study has evidenced significant dysbiosis regardless of administration route in mice [67]. Moreover, amoxicillin has been related to profound dysbiotic effects, with population richness and diversity significantly reduced, in orally treated pigs [62]. In Trial 1 we also observed that 2 and 7-days-old piglets from the Alpha farm presented lower abundances of pathogenic bacteria such as *Clostridium *sensu stricto* 1* and *Escherichia-Shigella*, whereas *Lactobacillus* counts were increased in Bravo piglets in most of the time-points (d2, d7, d21 and after weaning). Similar results have been reported in orally treated pigs with amoxicillin, where the antibiotic intervention decreased the abundance of *Lactobacillus* and other lactic acid bacteria [14, 62]. However, all these differences could had been due not only to the use of amoxicillin but also to differences in the composition of the oral solutions used in both farms that in Alpha not only included electrolytes but also a blend of mono, di and triglycerides of medium chain fatty acids, together a blend of acidifiers. With time and increasing age, the piglets from the Bravo farm ended up showing a more mature microbiota after weaning, with greater abundances of butyrogenic genera such as *Prevotella*, *Coprococcus*, *Faecalibacterium* and *Dorea*, among many others, as well as lower abundances of *Escherichia-Shigella* and *Fusobacterium* that could have been due to a lower selective pressure from antimicrobial prophylaxis. However, differences between farms after weaning in Trial 1 should be regarded with caution as the Alpha farm was sampled earlier than Bravo (7 vs. 14 days post-weaning) due to an imminent metaphylactic treatment of the piglets after the first signs of diarrhoea outbreak. This could explain the higher abundance of *Escherichia-Shigella* and *Fusobacterium* in Alpha piglets and the slower development of the fermentative bacteria.

In Trial 2, differences were also observed among rearing farms. In addition to the recurring *B. hyodysenteriae* problems in Echo and Foxtrot farms, which implied the consumption of medicated feed by the mothers, the main management difference consisted of the absence of the rehydrating solution in the piglets from the Foxtrot farm. Interestingly, in Foxtrot farm a decreased alpha diversity at day 2 and a marked increased abundance of *Enterobacteriaceae* both at 2 and 21 days of life were observed. Moreover, at 21 days of life, significant changes in other microbial groups were also observed in this farm, such as a greater abundance of *Enterococcaceae* and a lower relative abundance of *Lachnospiraceae* and *Fusobacteriaceae*. Although it is not possible to assure that the observed changes are due to either the initial antibiotic treatment or the re-hydrating solutions supplied during the first week of life of the piglets, it is evidenced that differential farm management guidelines, and particularly antimicrobial prophylaxis, can affect the development of the intestinal microbiota of the pig early in life.

Regarding the use of antibiotics in the mothers, results from Trial 2 showed ecological changes in the microbial communities of the piglet associated with this factor. The impact of maternal antibiotic treatment on the progeny has been a subject of study especially in the field of human medicine. It has been recently found that antibiotic use during pregnancy alters the commensal vaginal microbiota of women [68]. Moreover, amoxicillin administration during late gestation has been reported to impact both sow vaginal and faecal microbial diversities [69, 70]. Together, these results would suggest that maternal antibiotic treatment might influence the gut microbial colonization of the offspring through changing maternal microbiota composition. In addition, it is well known that maternal antibiotic residues can be transferred from mothers to their offspring via breastfeeding [71]. To date, very few studies have analysed the effect of the administration of antibiotics to mothers on their offspring, and there are especially few studies focused on the effects produced in their intestinal microbiota. Arnal et al. [70] reported a significant effect on the microbiota of the small intestine of the offspring but not of the colon. Moreover, some effects of maternal antibiotic treatment on the gut physiology and morphology of the offspring have been seen in early-life [69, 70, 72, 73]. In Trial 2 only farms Echo and Foxtrot gave the sows medicated feed during lactation. Two-day-old piglets from dams treated with medicated feed showed lower abundances of propionate- and butyrate-producing genera, such as *Phascolarctobacterium* and *Prevotella*, respectively. Lower abundances of *Enterococcus* were also observed. *Enterococcus* genera have recently gained attention due to their ability to produce bacteriocins recognized for their wide-range effectiveness on pathogenic and spoilage bacteria [74]. This is in consonance with the higher abundances of *Escherichia-Shigella* observed in piglets from dams treated with medicated feed. Piglets from treated dams also showed lower abundances of *Fusobacterium* at day 2 and 21 of life. Some authors have suggested that the abundance of *Fusobacteria*, is positively correlated with diarrheal swine diseases, such as the porcine epidemic diarrhoea and the new neonatal porcine diarrhoea [51, 52]. On day 21 of life, lower abundances of the acetate- and butyrate-producing genera *Rikenellaceae RC9 gut group* and *CAG-873*, respectively, were also observed in the piglets of mothers fed with medicated feed. Higher abundances of *Rikenellaceae RC9 gut group* are suggestive of a high level of functional redundancy for acetate in the swine gut microbiome [75]. A greater abundance of *Parabacteroides* was also observed in piglets from medicated dams. Higher abundances of *Parabacteroides* have been linked with lower BW and ADG [76, 77]. These findings suggest that maternal antibiotic treatment affected the gut microbiota of offspring through the transfer of maternal gut microbiota to newborn piglets, although it is difficult to specify whether the maternal antibiotic treatment affected the microbiota development of offspring in a direct or indirect process. Although some researchers have stated that maternal antibiotic treatment and early antibiotic administration affect the development of intestinal microbiota of the piglets, along with piglet mucosal tissue gene expression [11, 62, 63, 69, 78–81], further research is needed concerning the consequences of maternal antibiotic treatment with regards to the gut microbiome of their offspring.

Comparing both Trials (1 and 2), the process of the gut microbial colonization of piglets followed a similar evolution pattern between different farms, both in terms of species richness and microbiota diversity, which gradually increased in piglets with age, and in relation to the taxonomic groups involved in this process, despite the high individual variability observed at the earlier stages.

However, in line with the high variability expected at this early age [8], there is great variability among the six farms noticeable even from the phylum level (Additional file 1: Table S5). Thus, in Alpha and Bravo farms there are large initial abundances of Fusobacteria, while this difference seems to be occupied by Proteobacteria in the rest of the farms. Similarly, the phylum Firmicutes even doubles its relative abundance in the Alpha and Delta farms in comparison with the Foxtrot farm and drastic changes are also observed in the Bacteroidetes group. Similar results are observed at the family level, with large differences among farms and piglets of the same age. While in Charlie, Delta, Echo, and Foxtrot the predominant family is *Enterobacteriaceae*, in Alpha and Bravo the *Fusobacteriaceae* family also plays an important role, becoming the predominant family in the case of the Alpha farm. Concerning Clostridia, *Clostridicaeae* is observed in all farms with an average relative abundance of around 20%, with quite a similarity between different farms. Therefore, although it is true that the predominant groups in two-day-old piglets are *Enterobacteriaceae* and *Clostridiaceae*, there is some controversy with the *Fusobacteriaceae* family. The high abundance of *Fusobacteria* observed in Alpha and Bravo farms (Trial 1) during the first days of life has also been reported by several other studies [21, 32, 38–40]. However, some studies have not reported the presence of this bacterial group at all [16, 35]. Such differences in taxonomic abundance could, to some extent, be due to uncontrolled environmental differences between Trials (each trial was run in a different time window) but also to methodological bias. Despite all samplings being carried out using the same procedures and samples analyzed in the same lab with the same methodology, certain batch effect cannot be discarded.

Regarding the distinctive higher abundance of Fusobacteria in Alpha and Bravo farms (Additional file 1: Table S6) it is interesting to highlight that higher abundances of Fusobacteria were associated with a greater abundance of minority genera representing less than 1% of the total (all together close to 30% of relative abundance vs. 8.9% in farms form Trial 2). These results would suggest *Fusobacterium* as a possible indicator of a more diverse early colonization. A greater evenness between minor bacterial groups could be considered as beneficial since it could represent an increased capacity for adaptation and resilience. Actually, in Charlie, Delta, Echo, and Foxtrot farms a very high abundance of *Escherichia-Shigella* is observed (around 40%, although reaching up to 71% on the Foxtrot farm) whereas, in Alpha and Bravo farms, the *Escherichia-Shigella*, *Bacteroides,* and *Fusobacterium* genera share similar abundances (around 10%, without any of them standing out excessively). However, despite these results, *Fusobacterium* has been typically associated with diarrhoea and gut inflammation [52, 82, 83]. Moreover, it should be noted that within Trial 2 farms, Echo and Foxtrot showed particularly low relative abundances of *Fusobacterium,* being those the farms in which antibiotics were administered to the sows. Actually, *Fusobacterium* has been reported to reduce significantly after an antibiotic treatment [52]. In any case, all these data would point out *Fusobacterium* as a possible microbial community marker that would deserve more attention in future studies.

There are very few works, that have tried to assess the impact of the environment or rearing practices on the establishment of the intestinal microbiota of the piglets after birth in commercial conditions. Recently, Lührmann et al. [84] analyzing the faecal microbiota from sows and their piglets, at two time points each, in 20 German farms (sows ante- and postpartum and piglets before and after weaning) were also able to identify differences related to farms, particularly when they analyze the family-unit mother-piglet, suggesting the existence of a farm-specific microbiome of sows and piglets. Despite these encouraging results, authors also recognize the difficulty of making a comprehensive analysis of the role that feeding, housing, and management could have in such definition. Undoubtedly, more studies are needed to fully elucidate the factors that could condition such farm-specific microbiomes and their role in the health and performance of piglets.

## Conclusions

Taken together, the present study confirms and refines the knowledge about microbiome development during early-life stages in piglets. The initial colonization characterized by bacteria belonging to the *Clostridiaceace*, *Enterobacteriaceae* and *Fusobacteriaceae* families is progressively replaced by fermenting bacteria, essentially the acetate, propionate and butyrate-producing microorganisms. Moreover, this study evidences that the rearing farm and particularly the use of antimicrobials in the sow can have a significative impact on this process. Feeding antibiotics to the mothers can induce ecological changes in the piglets without changes in microbiota richness or diversity. Significant shifts can be observed in particular microbial groups, and between them, lower abundances of *Fusobacterium* in those piglets from medicated sows. This work also evidences that the use of oral rehydrating solutions can modulate the piglets’ microbiota with significant reductions in the abundance of the *Enterobacteriaceae* family. All in all, our results demonstrate that the farm and the rearing practices, particularly the use of antimicrobials in the mothers, can determine structural and taxonomic changes in the gut microbiota of the young piglet. More studies will be needed to fully understand the underlying phenomena before designing intervention strategies addressed to improve the resilience of the piglet intestinal ecosystem.

## Supplementary Information

**Additional file 1.** Supplementary Tables and Figure.

## Data Availability

The raw sequencing data employed in this article has been submitted to the NCBI’s sequence read archive (https://www.ncbi.nlm.nih.gov/sra); BioProject: PRJNA800765.

## Abbreviations

ANOSIM: Analysis of similarities
ANOVA: Analysis of variance
APP: Actinobacillus pleuropneumonia
ASV: Amplicon sequence variant
BW: Body weight
CP: Crude protein
CSS: Cumulative sum scaling
DADA2: Divisive amplicon denoising algorithm 2
DNA: Deoxyribonucleic acid
MHA: Methionine hydroxy analogue
NMDS: Non-metric multidimensional scaling
PERMANOVA: Permutational multivariate analysis of variance
PRRS: Porcine Reproductive and Respiratory Syndrome
RA: Relative abundance
SID: Standardized ileal digestible
STTD: Standardized total tract digestible
ZnO: Zinc oxide
