# Supplementary material for: An insight into the commercial piglet’s microbial gut colonization: from birth towards weaning
Source: Anim Microbiome. 2022 Dec 26;4:68. doi: 10.1186/s42523-022-00221-9 (PMC9791761; doi:10.1186/s42523-022-00221-9)
Supplement: Supplementary file 1 — Additional file 1. Supplementary Tables and Figure. [file 42523_2022_221_MOESM1_ESM.pdf]

Additional files

Additional file 1: Table S1. Sow standard lactation feed formulas and estimated nutrient content of the experimental basal diets.

**Ingredients, %**	
Barley (10% CP)	32.07
Corn	17.29
Wheat bran	18.00
Sunflower (28% CP)	10.50
Soybean expeller (44% CP)	11.00
Lard	2.00
Animal fat	4.00
Hydrolysed mucosa	2.00
Calcium carbonate	1.68
Dicalcium phosphate	0.32
Salt	0.27
Lysine sulphate 70%	0.39
Methionine hydroxy analogue	0.03
L-Threonine	0.07
L-Valine 1814	0.02
Vitamin Mineral premix	0.30
Choline chloride 75%	0.04
Liquid 6-phytase	0.02
Antibiotic 220 g/kg premix	None or 0.06[1]
**Nutritional composition (as fed basis)**	
Metabolizable energy content, kcal/kg	3117
Dry matter, %	89.10
Starch, %	31.43
Neutral detergent fibre, %	20.04
Acid detergent fibre, %	8.96
Fat, %	8.94
CP, %	16.09
Lysine, %	0.93
Methionine + Cystine, %	0.57
Threonine, %	0.64
Tryptophan, %	0.20
Ash, %	5.66
Calcium, %	0.83
Total phosphorus, %	0.57

[1] In the second Trial, sows from **Echo** and **Foxtrot** farms received medicated feed with an antibiotic premix (600 ppm, **ABF**), whereas **Charlie** and **Delta** sows did not receive any antimicrobial treatment (non-medicated feed, **NMF**).

Additional file 1: Table S2. Impact of age and farm on piglet faecal microbiota biodiversity. (Trial 1). Data are expressed as mean ± standard error. WP = Weaned piglets.

	Observed species	Chao1	Shannon	Simpson	Inverse Simpson
Age					
d2	905 ± 97.47	908 ± 97.42	5.90 ± 0.109	0.995 ± 0.001	243 ± 21.97
d7	1151 ± 120.4	1154 ± 120.91	6.33 ± 0.106	0.997 ± 0	392 ± 41.76
d14	1413 ± 145.71	1419 ± 145.49	6.63 ± 0.09	0.998 ± 0	532 ± 48.07
d21	1543 ± 212.44	1548 ± 213.02	6.64 ± 0.094	0.998 ± 0	543 ± 61.45
WP	2012 ± 304.66	2020 ± 304.43	7.01 ± 0.137	0.999 ± 0	914 ± 107.69
Farm					
Alpha	903 ± 65.89	909 ± 66.33	6.24 ± 0.064	0.997 ± 0	389 ± 23.4
Bravo	1906 ± 139.8	1911 ± 139.89	6.77 ± 0.088	0.998 ± 0	662 ± 61.75
P-value					
Age	<0.001	<0.001	<0.001	<0.001	<0.001
Farm	<0.001	<0.001	<0.001	0.018	<0.001
Age:Farm	0.005	0.005	0.006	0.354	<0.001

Additional file 1: Table S3. Impact of age in alpha diversity between farms in Trial 1.

Observed species

	d2	d7	d14	d21	WP
Alpha	659.00	736.70	896.40	1204.30	1023.30
Bravo	1151.70	1566.40	1930.60	1881.90	3001.40
SEM	97.469	120.400	145.714	212.443	304.661
P-value	0.007	<0.001	<0.001	0.113	<0.001

Chao1

	d2	d7	d14	d21	WP
Alpha	661.78	738.38	902.37	1210.67	1032.38
Bravo	1155.07	1571.32	1936.06	1886.22	3008.17
SEM	97.415	120.911	145.488	213.016	304.433
P-value	0.007	<0.001	<0.001	0.115	<0.001

Shannon

	d2	d7	d14	d21	WP
Alpha	5.86	6.03	6.33	6.46	6.50
Bravo	5.95	6.62	6.92	6.83	7.51
SEM	0.109	0.106	0.090	0.094	0.137
P-value	0.699	0.002	<0.001	0.043	<0.001

Simpson

	d2	d7	d14	d21	WP
Alpha	0.995	0.996	0.997	0.998	0.998
Bravo	0.995	0.998	0.998	0.998	0.999
SEM	0.001	0.000	0.000	0.000	0.000
P-value	0.822	0.024	0.006	0.371	<0.001

Inverse Simpson

	d2	d7	d14	d21	WP
Alpha	252.10	298.85	417.49	441.33	535.29
Bravo	235.53	486.37	648.15	645.31	1294.50
SEM	21.969	41.758	48.069	61.452	107.694
P-value	0.717	0.020	0.012	0.098	<0.001

Additional file 1: Table S4. Impact of age, farm and in-feed antibiotic supplementation of sows, on piglet faecal microbiota biodiversity. (Trial 2). Data are expressed as mean ± standard error.

	Observed species	Chao1	Shannon	Simpson	Inverse Simpson
Age					
d2	778 ± 49.15	781 ± 49.21	5.35 ± 0.101	0.990 ± 0.001	154 ± 14.90
d21	1834 ± 75.22	1840 ± 75.24	6.95 ± 0.060	0.998 ± 0.000	773 ± 44.98
Farm					
Charlie	1430[a] ± 121.73	1434[a] ± 121.79	6.33[a] ± 0.174	0.995 ± 0.001	524 ± 73.86
Delta	1187[b] ± 122.69	1192[b] ± 123.20	6.14[ab] ± 0.172	0.995 ± 0.001	411 ± 63.42
Echo	1427[a] ± 159.22	1431[a] ± 159.21	6.31[a] ± 0.189	0.995 ± 0.001	495 ± 83.88
Foxtrot	1170[b] ± 146.6	1176[b] ± 146.96	5.82[b] ± 0.239	0.992 ± 0.002	417 ± 87.85
Dietary treatment (use of AB in the sow)					
NMF	1318 ± 87.44	1322 ± 87.61	6.24 ± 0.123	0.995 ± 0.001	472 ± 49.54
ABF	1293 ± 108.39	1298 ± 108.51	6.05 ± 0.156	0.993 ± 0.001	454 ± 60.53
P-value					
Age	**<0.001**	**<0.001**	**<0.001**	**<0.001**	**<0.001**
Farm	**0.033**	**0.034**	**0.027**	0.086	0.252
Use of AB	0.785	0.791	0.108	0.075	0.718
Age:Farm	0.148	0.150	0.297	0.229	0.501
Age:AB	0.137	0.139	0.231	0.209	0.766

Additional file 1: Table S5. Relative abundances (RAB) of the main phyla (RAB greater than 0.1%) and families (RAB greater than 0.5%) in the two-day-old piglets, ordered from highest to lowest abundance in relation to the mean. The mean of each taxonomic group with its standard deviation is also indicated in the last column of the table. The relative abundance of the rest of the taxonomic groups not included in the table have been grouped and are shown in the label "Other" with its respective percentage.

	Alpha	Bravo	Charlie	Delta	Echo	Foxtrot	Global mean
Phylum							
Proteobacteria	24.8	34.5	49.1	51.0	56.0	76.0	48.6 ± 17.76
Firmicutes	39.7	34.3	35.4	39.8	26.9	20.3	32.7 ± 7.73
Bacteroidetes	12.1	16.5	7.24	3.26	15.2	3.02	9.55 ± 5.886
Fusobacteria	22.5	14.2	6.33	3.18	0.92	0.37	7.91 ± 8.745
Actinobacteria	0.36	0.15	0.94	0.87	0.51	0.20	0.51 ± 0.336
Other (< 0.1%)	0.02	0.22	0.16	0.19	0.06	0.01	0.11 ± 0.091
Family							
Enterobacteriaceae	17.9	31.3	37.3	36.0	43.2	71.8	39.6 ± 17.93
Clostridiaceae	22.4	14.7	24.9	24.8	13.6	16.3	19.4 ± 5.16
Fusobacteriaceae	22.5	14.2	6.30	3.16	0.92	0.37	7.89 ± 8.735
Bacteroidaceae	7.43	13.8	4.03	1.73	14.3	2.83	7.36 ± 5.541
Lachnospiraceae	7.59	8.14	1.67	4.51	3.92	1.33	4.53 ± 2.872
Streptococcaceae	4.74	2.08	3.22	4.40	3.19	1.49	3.19 ± 1.264
Pasteurellaceae	6.07	2.94	1.03	2.33	3.36	2.55	3.05 ± 1.678
Alcaligenaceae	0.08	0.02	6.93	7.57	2.49	0.56	2.94 ± 3.462
Prevotellaceae	4.23	2.02	1.55	0.67	0.16	0.04	1.45 ± 1.568
Veillonellaceae	1.24	2.69	0.56	1.27	1.82	0.19	1.29 ± 0.895
Burkholderiaceae	0.09	0.00	0.23	1.14	4.47	0.54	1.08 ± 1.713
Lactobacillaceae	1.00	2.94	0.79	0.61	0.45	0.23	1.00 ± 0.985
Moraxellaceae	0.17	0.06	1.14	2.84	0.90	0.11	0.87 ± 1.064
Peptostreptococcaceae	0.42	0.26	0.67	0.40	1.47	0.16	0.56 ± 0.479
Enterococcaceae	0.32	1.39	0.35	0.80	0.14	0.10	0.52 ± 0.494
Sutterellaceae	0.36	0.13	1.47	0.35	0.07	0.21	0.43 ± 0.52
Oscillospiraceae	0.33	0.31	0.53	0.72	0.30	0.03	0.37 ± 0.233
Acidaminococcaceae	0.18	0.77	0.34	0.21	0.05	0.07	0.27 ± 0.267
Butyricicoccaceae	0.87	0.19	0.10	0.08	0.14	0.07	0.24 ± 0.311
Other (< 0.5%)	2.08	2.03	6.86	6.45	5.06	1.00	3.92 ± 2.523

Additional file 1: Table S6. Relative abundances of the main genera (RAB greater than 1%) in the two-day-old piglets, ordered from highest to lowest abundance in relation to the mean. The mean of each taxonomic group with its standard deviation is also indicated in the last column of the table. The relative abundance of the rest of the taxonomic groups not included in the table have been grouped and are shown in the label "Other" with its respective percentage.

	Alpha	Bravo	Charlie	Delta	Echo	Foxtrot	Global mean
Escherichia-Shigella	10.4	10.2	37.1	35.6	42.5	71.7	34.6 ± 22.91
Clostridium sensu stricto 1	5.45	2.64	24.7	24.6	13.2	16.1	14.4 ± 9.30
Bacteroides	11.3	12.5	4.03	1.73	14.3	2.83	7.78 ± 5.517
Fusobacterium	9.76	10.3	6.26	3.13	0.91	0.37	5.13 ± 4.34
Alcaligenes	0.00	0.02	6.92	7.56	2.47	0.55	2.92 ± 3.474
Streptococcus	1.13	1.64	3.20	4.35	3.18	1.48	2.49 ± 1.269
Lactobacillus	1.66	6.76	0.79	0.61	0.45	0.23	1.75 ± 2.504
Actinobacillus	0.84	1.60	0.85	1.76	2.54	2.33	1.65 ± 0.715
Prevotella	0.63	5.05	1.02	0.31	0.03	0.02	1.18 ± 1.934
UCG-002	4.75	1.26	0.22	0.28	0.07	0.01	1.1 ± 1.848
Ralstonia	0.09	0.00	0.23	1.14	4.47	0.54	1.08 ± 1.712
Lachnospiraceae UCG-004	3.56	0.98	0.16	0.29	0.17	0.06	0.87 ± 1.361
Veillonella	0.37	1.06	0.43	1.25	1.78	0.19	0.84 ± 0.62
Phascolarctobacterium	1.71	2.58	0.34	0.20	0.05	0.07	0.83 ± 1.066
Enterococcus	2.70	0.63	0.35	0.80	0.14	0.10	0.79 ± 0.977
UCG-005	2.71	1.41	0.15	0.12	0.12	0.01	0.75 ± 1.093
Dorea	0.97	1.11	0.20	0.65	0.97	0.07	0.66 ± 0.436
Lachnoclostridium	0.67	1.98	0.16	0.50	0.52	0.15	0.66 ± 0.678
Rikenellaceae RC9 gut group	1.98	1.35	0.22	0.22	0.04	0.01	0.64 ± 0.826
Acinetobacter	0.00	0.01	0.79	2.62	0.21	0.03	0.61 ± 1.032
Campylobacter	2.32	1.07	0.01	0.00	0.01	0.00	0.57 ± 0.957
Christensenellaceae R-7 group	2.04	0.59	0.15	0.20	0.06	0.01	0.51 ± 0.777
Sutterella	0.58	0.25	1.47	0.35	0.05	0.21	0.48 ± 0.513
Treponema	0.94	1.38	0.08	0.06	0.04	0.00	0.42 ± 0.595
Lachnospiraceae NK4A136 group	2.04	0.19	0.02	0.04	0.01	0.00	0.38 ± 0.813
NK4A214 group	0.83	1.12	0.07	0.13	0.08	0.00	0.37 ± 0.478
Subdoligranulum	0.28	1.40	0.09	0.19	0.04	0.00	0.33 ± 0.532
Prevotellaceae NK3B31 group	0.15	1.17	0.20	0.12	0.03	0.01	0.28 ± 0.443
Sphaerochaeta	1.13	0.18	0.01	0.03	0.01	0.00	0.23 ± 0.446
Other (< 1%)	29.0	29.6	9.8	11.1	11.6	2.94	15.7 ± 11.01

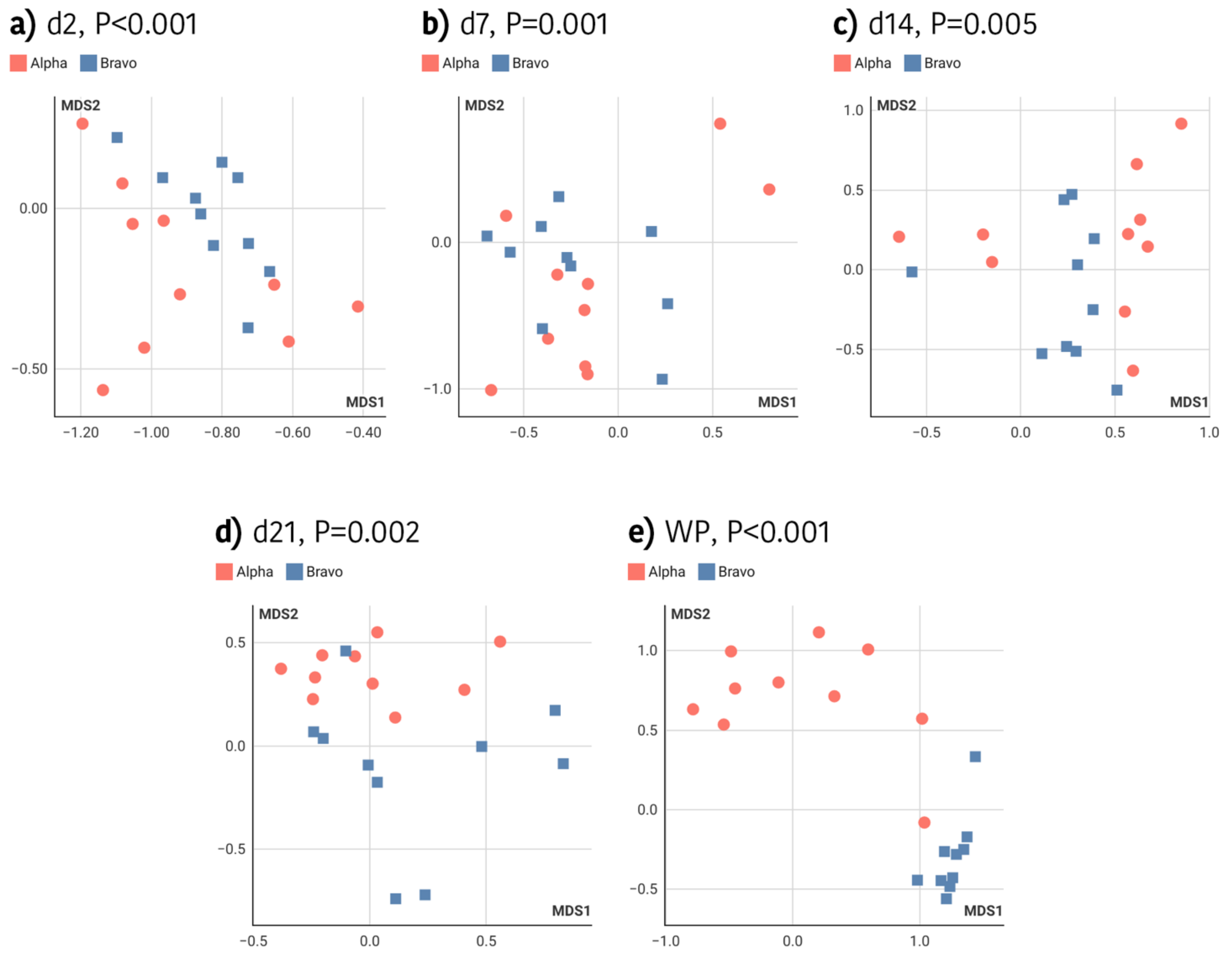

Additional file 1. Figure S1. NMDS of the relative abundances of ASV during trial 1 for each sampling day. Five additional permutational analysis of variance (PERMANOVA) were performed. All comparisons between **Alpha** and **Bravo** farms were significant at each sampling age.

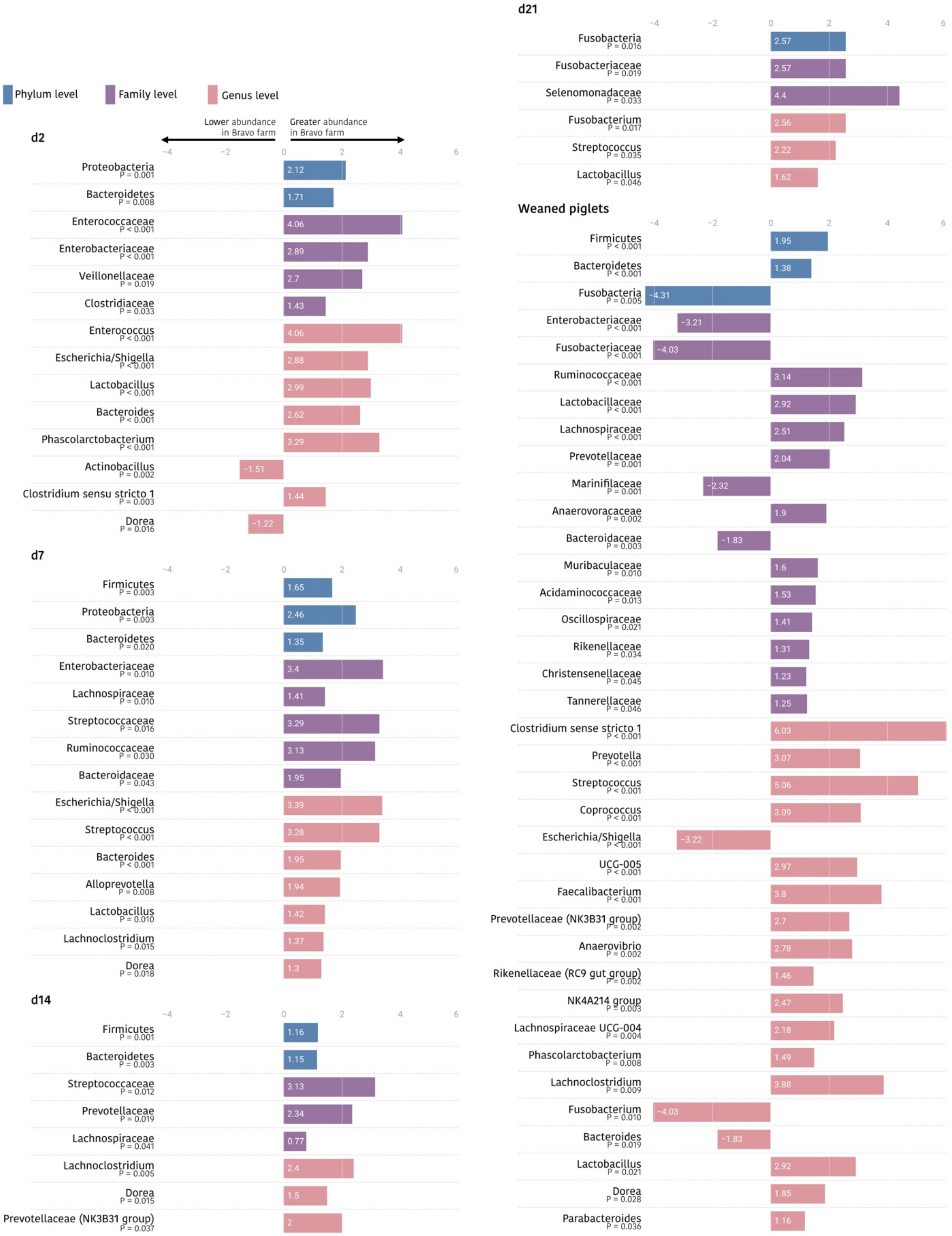

Additional file 1. Figure S2. Ln changes in taxa promoted by farm origin (**Bravo** vs **Alpha**; ln change and P-value<0.05) at phylum, family and genus level in the microbiota of piglets sampled on days 2, 7, 14 and 21 of lactation and 14 days post-weaning (7 days in **Alpha** farm). Piglets were weaned at 21 days of age. Positive values and negative values indicate higher and lower abundance, respectively, in piglets from Bravo farm. Taxa are sorted by level of significance (from higher to lower). Only taxa with relative abundances higher to 1% are included in the figure. The presented differences are based only on taxa detected in at least half of the samples per sampling. Figure created with the online open-source tool Datawrapper (http://datawrapper.de).
